# New Helvolic Acid Derivatives with Antibacterial Activities from *Sarocladium oryzae* DX-THL3, an Endophytic Fungus from Dongxiang Wild Rice (*Oryza rufipogon* Griff.)

**DOI:** 10.3390/molecules26071828

**Published:** 2021-03-24

**Authors:** Zhi-Bin Zhang, Si-Yao Du, Bo Ji, Chang-Jiu Ji, Yi-Wen Xiao, Ri-Ming Yan, Du Zhu

**Affiliations:** 1 Key Laboratory of Protection and Utilization of Subtropical Plant Resources of Jiangxi Province, College of Life Science, Jiangxi Normal University, Nanchang 330022, China; zzbbio@jxnu.edu.cn (Z.-B.Z.); dusiyao2628@163.com (S.-Y.D.); xyw1152858687@163.com (Y.-W.X.); rimingyan@163.com (R.-M.Y.); 2 College of Life Science, Jiangxi Science and Technology Normal University, Nanchang 330013, China; jibo_ky@163.com; 3 College of Chemistry and Biological Engineering, Yichun University, Yichun 336000, China

**Keywords:** endophytic fungus, *Sarocladium oryzae*, helvolic acid derivatives, antibacterial activity, SAR

## Abstract

Three new helvolic acid derivatives (named sarocladilactone A (**1**), sarocladilactone B (**2**) and sarocladic acid A (**3a**)), together with five known compounds (6,16-diacetoxy-25-hy- droxy-3,7-dioxy-29-nordammara-1,17(20)-dien-21-oic acid (**3b**), helvolic acid (**4**), helvolinic acid (**5**), 6-desacetoxy-helvolic acid (**6**) and 1,2-dihydrohelvolic acid (**7**)), were isolated from the endophytic fungus DX-THL3, obtained from the leaf of Dongxiang wild rice (*Oryza rufipogon* Griff.). The structures of the new compounds were elucidated via HR-MS, extensive 1D and 2D NMR analysis and comparison with reported data. Compounds **1**, **2**, **4**, **5**, **6** and **7** exhibited potent antibacterial activities. In particular, sarocladilactone B (**2**), helvolinic acid (**5**) and 6-desacetoxy-helvolic acid (**6**) exhibited strongly *Staphylococcus aureus* inhibitory activity with minimum inhibitory concentration (MIC) values of 4, 1 and 4 μg/mL, respectively. The structure–activity relationship (SAR) of these compounds was primarily summarized.

## 1. Introduction

Endophytic fungi have been recognized as a rich source of potent bioactive natural products with diverse structural groups, and a large number of metabolites, such as alkaloids, steroids, terpenoids, peptides, polyketones, flavonoids and xanthones, are continuously being isolated from them [1,2]. The various natural products possess unique structures and bioactivities, thus representing a huge reservoir that offers an enormous amount of potential for exploitation in the agricultural and industrial areas [3].

Strain DX-THL3 was isolated from the leaf of Dongxiang wild rice (*Oryza rufipogon* Griff.) in the Jiangxi Province of China, and identified as *Sarocladium oryzae* [4]. *S. oryzae* is an Ascomycetes fungus that causes sheath rot disease in rice [5]. Several studies have reported that *S. oryzae* produces helvolic acid and cerulenin secondary metabolites, which have been reported as phytotoxins [6,7]. Helvolic acid was originally isolated from the culture filtrates of *Aspergillus fumigatus mut. helvola* Yuill [8], and the confirmation of its structure was completed by Shigeo Iwasaki [9]. Nowadays, helvolic acid is a representative fusidane-type antibiotic, which exhibits potent activity against Gram-positive bacteria [10]. Fusidane-type antibiotics have recently attracted renewed attention for their lack of cross resistance with other commonly used antibiotics [11]. Up until now, helvolic acid was generally found in marine-derived or terrestrial fungi of the *Aspergillus* spp., and in other endophytic fungi, such as *Fusarium* sp. [12], *Penicillium* sp. [13] and *Sarocladium* sp. [6]. However, most of the newly found helvolic acid derivatives were reported from metabolites of *Aspergillus* spp. [14,15,16,17].

**Figure 1:**
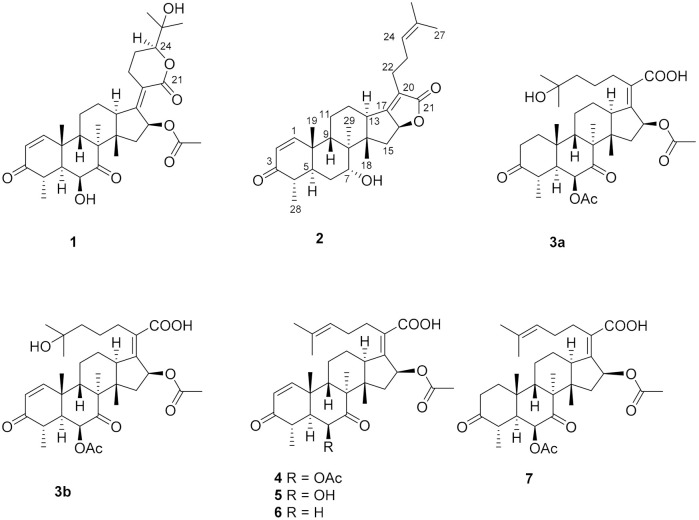
The chemical structures of compounds **1**–**7**.

In the course of our ongoing research, DX-THL3 expressed antimicrobial activities and activities against phytopathogens [18]. Thus, it attracted us to find new chemical entities and potent bioactive products from DX-THL3. Subsequent chemical investigation of the EtOAc extract of the fermentation broth led to the identification of three new helvolic acid derivatives (**1**, **2** and **3a**), together with five known compounds (**3b**, **4**–**7**). Among them, sarocladilactone A (**1**), sarocladilactone B (**2**), helvolic acid (**4**), 6-deacetoxy helvolic acid (**5**), helvolinic acid (**6**) and 1,2-dihydrohelvolic acid (**7**) showed potent antimicrobial activities against *S. aureus.* Herein, the isolation, structure elucidation and bioactivities of these compounds are described (Figure 1).

## 2. Results and Discussion

### 2.1. Identification of Fungus Identification

The strain of DX-THL3 was identifed as *Sarocladium oryzae* (located in GenBank under the accession number KF558875) based on DNA amplification and the internal transcribed spacer (ITS) sequence (Figure S1).

### 2.2. Structure Elucidation

**Figure 2:**
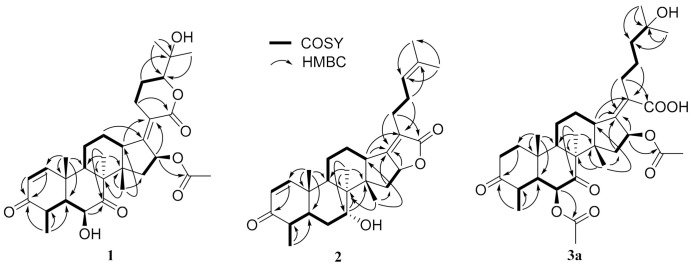
The key COSY (bold lines) and HMBC (arrows) correlations of compounds **1**, **2** and **3a**.

Compound **1**, a white powder, exhibited a pseudomolecular ion peak at *m*/*z* 543.2961 (M + H)^+^ in the HRESIMS spectrum, indicating a molecule formula of C_31_H_42_O_8_ with 11 degrees of unsaturation. The ^1^H NMR spectrum showed six singlet methyl peaks and a doublet methyl peak, and three oxygenated sp^3^ methine groups of H-6, H-16, H-24 exhibited a broad singlet at δ_H_ 4.03, a broad doublet at δ_H_ 5.85, and a double-doublet at δ_H_ 4.09, respectively. The ^13^C NMR spectrum (Table 1) showed 7 methyl groups (including an acetyl group), 5 sp^3^ methene groups, 9 methine groups (including 3 oxygenated sp^3^ methine groups, a pair of cis-double bond at δ_C_ 157.8 and 127.7 and two doublet peaks at δ_H_ 7.30 and 5.85 with a coupling constant of 10.0 Hz), 10 quaternary carbons (including two keto carbonyls at δ_C_ 213.2 and 202.1, two ester or lactone carbonyls at δ_C_ 169.9 and 164.8, two olefinic carbons at δ_C_ 155.0 and 125.1 and an oxygenated sp^3^ carbon at δ_C_ 71.8), all of which suggest that compound **1** has a pentacyclic ring system, and both an α,β-unsaturated ketone and an α,β-unsaturated lactone unit exist. By comparing the NMR data, it closely resembles that of the reported 24-epi-6β,16β-diacetoxy-25- hydroxy-3,7-dioxo-29nordamara-1,17(20)- diene-21,24-lactone [14,15] except for an acetyl deficiency at C-6, resulting in a remarkable downshift of 4.8 ppm for C-7 (δ_C_ 213.2), a slight upshift of 0.8~0.9 ppm between C-28 and C-29 and a prominent upshift of 1.22 ppm for H-6 (δ_H_ 4.03), together with a downshift of 0.25 ppm for H-4 (δ_H_ 3.02). The correlation of H-16 to the acetyl (δ_C_ 169.9) in the HMBC spectrum is another piece of collateral evidence supported by the acetyl deficiency at C-6 (Figure 2).

**Figure 3:**
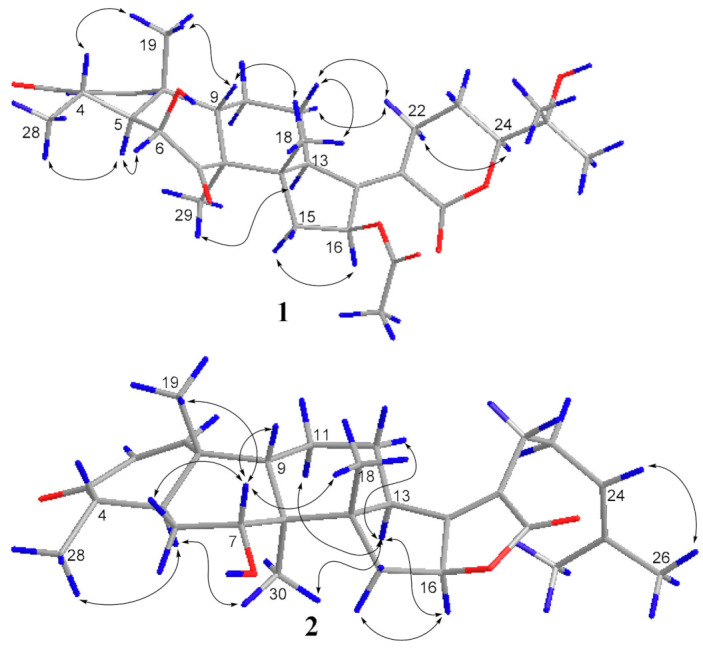
The key ROESY correlations of compound **1** and **2**.

The ROESY spectrum of **1** also showed close resemblance to above reported compound for the correlations between the equatorial proton H-22a (δ_H_ 3.11) and the equatorial proton H-12a (δ_H_ 2.49) and the axial proton H-12b (δ_H_ 1.82), and the correlation between H-24 (δ_H_ 4.09) and the axial proton H-22b (δ_H_ 2.43) (Figure 3), while no correlation was found between the two axial protons H-22b and H-12b, which supported the α-oriented configuration of the axial proton H-24. Hence, the structure of **1** was unambiguously assigned as 24-epi-16β-acetoxy-6β,25-dihydroxy-3,7-dioxo-29-nordamara-1,17(20)-diene-21,24-lactone, and given the trivial name sarocladilactone A.

Compound **2** is a white powder with a slight impurity. Its molecular formula was determined to be C_29_H_42_O_4_ from HRESIMS peaks at *m*/*z* 453.3016 (M + H)^+^ (cacld 453.3005) and *m*/*z* 475.2830 (M + Na)^+^ (cacld 475.2824), requiring 10 degrees of unsaturation. Via extensive analysis of HSQC and ^1^H-^1^H COSY spectra, the impurity signals in ^1^H NMR and ^13^C NMR spectra were excluded, and the primary remaining NMR chemical shifts of compound **2** were recognized, as shown in Table 1. The ^13^C NMR spectrum exhibited six methyl groups, six methene groups, nine methine groups and eight quaternary carbons and revealed the presence of an α,β-unsaturated ketone, an α,β-unsaturated lactone and a trisubstituted ethenyl moetity, which resemble those of 16-*O*-deacetylhelvolic acid 21,16-lactone [15]. Moreover, except for the presence of double-doublet peaks at δ_H_ 4.99 (*J* = 11.1, 4.7 Hz, H-16), δ_C_ 84.1 (C-16), additional double-doublet peaks at δ_H_ 4.04 (*J* = 8.4, 4.1 Hz, H-7) and δ_C_ 70.6 (C-7) were connected with an asymmetric CH_2_ (C-6), which is similar to the configuration of maunakeanolic acid A [16], suggesting that compound **2** is a 6-deacetoxyl and 7-hydroxyl substituted derivative of 16-*O*-deacetylhelvolic acid 21,16-lactone and can be further identified via HMBC correlations (Figure 2). The α-orientation of the 7-hydroxyl group was clearly determined by the observed correlations between axial protons H-7/H-9, H-7/CH_3_-18 and H-7/CH_3_-19 in the ROESY spectrum (Figure 3). Thus, compound **2** was elucidated as 6-deacetoxyl-7α-hydroxyl-16-*O*-deacetylhelvolic acid 21,16-lactone and given the trivial name sarocladilactone B.

Compounds **3a** and **3b** manifested as a white mixture solid, and the amount of compound **3a** was a little higher than that of **3b**. The negative HRESIMS showed a pair of molecular ions (588.3439, 586.3267) and their quasimolecular ions (587.3377, 585.3232), indicating they share the same skeleton with a difference of 2 Da, and the formulae of C_33_H_48_O_9_/C_33_H_46_O_9_ were given. From the ^1^H, ^13^C NMR and DEPT spectra, the two compounds are also helvolic acid derivatives (Table 1). Combination with extensive 2D NMR analysis, the signals were finely sorted and assigned. The result implied that the difference between **3a** and **3b** lies only in the unsaturation of C_1_-C_2_. Additionally, they have a distinctly oxygenated quaternary carbon at δ_C_ 69.8 (C-25), and two acetoxyl group, two keto carbonyl, one carboxyl, and one or two olefinic bond, and compound 3b is identical to the reported (4*S*,5*S*,6*S*,8*S*,9*S*,10*R*,13*R*,14*S*,16*S*,17*Z*) 6,16-diacetoxy-25-hydroxy-3,7-dioxy-29-nordammara-1,17 (20)-dien-21-oic acid, which was confirmed via the ^1^H-^1^H COSY, HMBC and ROESY spectra [13]. Thus, compound **3a** was elucidated as a 1,2-dihydro derivative of **3b**, and given the trivial name sarocladic acid A (Figure 2 and Figure 3).

Compounds **5** and **6** were firstly reported as hydrolyzed products from helvolic acid (**4**), named helvolinic acid and 6-deacetoxyhelvolic acid, respectively. They exhibit a similar antibiotic spectrum. Meanwhile, they were separated from a cuture broth of *Cephalosporium caerulens*, indicated they are precursors of **4** [19], with no detailed NMR spectroscopic data. Herein, the ^1^H and ^13^C NMR data of compounds **5** and **6** were fully elucidated and assigned.

Compound **7**, named 1,2-dihydrohelvolic acid, was previously reported by Zaman [16] and Lee [20], respectively, and only the NMR data recorded in pyridine-*d*_5_ are displayed, while the data recorded in methanol-*d*_4_ are not available. Herein, the significant ^1^H and ^13^C NMR data are listed in Table 2.

### 2.3. Characterization of Compounds **1**,**2** and **3a**

Sarocladilactone A (**1**): white amorphous powder; [α]^25^_D_ −20 (c 0.1, MeOH); ^1^H NMR and ^13^C NMR data, see Table 1; HRESIMS *m*/*z*: 543.2961 (M + H)^+^ (calcd for C_29_H_41_O_4_: 543.2958).

Sarocladilactone B (**2**): white amorphous powder; [α]^25^_D_ −52 (c 0.1, MeOH); ^1^H NMR and ^13^C NMR data, see Table 1; HRESIMS *m*/*z*: 453.3016 (M + H)^+^ (calcd for C_29_H_41_O_4_: 453.3005).

Sarocladic acid A (**3a**): white amorphous powder; ^1^H NMR and ^13^C NMR data, see Table 1; HRESIMS *m*/*z*: 588.3439 (M + H)^+^ (calcd for C_33_H_48_O_9_: 588.3298).

### 2.4. Biological Activities

The antibacterial activities of the isolated compounds **1**–**7** (compounds **3a** and **3b** were not tested due to the presence of mixtures) against *Staphylococcus aureus*, *Bacillus subtilis*, *Escherichia coli* and *Xanthomonas oryzae pv.oryzicola* were evaluated using the 2-fold dilution assay. Compounds **1**, **2**, **4**, **5**, **6** and **7** showed antibacterial activity against *S. aureus* with MIC values of 64, 4, 8, 1, 4 and 16 μg/mL (Table 3), respectively (with tobramycin as the positive control, MIC 1 μg/mL), while compound **5** also showed antibacterial activity against *B. subtilis* with an MIC value of 64 μg/mL (with tobramycin as the positive control, MIC 64 μg/mL). Compounds **2**, **5** and **7** showed some potent antibacterial activity against *E. coli* with MIC 64 μg/mL (Table 3). All of the compounds were inactive against *X. oryzae pv.oryzicola* at 128 μg/mL. It was reported that helvolic acid and its derivatives exhibited better antibacterial activities, mainly against Gram-positive bacteria [21,22].

On the basis of the structural differences between compounds **4**–**7** and compounds **1** and **2**, comparing the MIC values of compounds **4** and **7**, it can be concluded that the α,β-unsaturated ketone unit in ring-A contributes the activity about 2-fold. The corresponding acetoxy substituent at C-6 in helvolic acid was replaced by a hydroxyl group (compound **5**), for which activity is significantly increased 8-fold versus compound **4**. The acetoxyl deficiency at C-6 of helvolic acid (compound **6**) is significantly increased 2-fold versus compound **4**.

As for compound **2**, there is a C-21/C-16 lactone ring present, which will theoretically lessen the antibacterial activity [15], while for an α-orientation hydroxyl substitution at C-7 instead of an ortho–hydroxy carbonyl in Ring-B, the MIC value is less than that of compound **4**.

To summarize, regarding the antibacterial structure–activity relationship (SAR) of helvolic acid and its derivatives, it can be concluded that the presence of a C-21/C-16 lactone ring and a C-21/C-24 lactone ring significantly reduced the antibacterial activity. Remarkably, compound **5**, with a hydroxyl group replacing the corresponding acetoxy substituent at C-6 in helvolic acid, exhibited stronger antibacterial activity against *S. aureus* than the antibacterial drug helvolic acid.

## 3. Materials and Methods

### 3.1. General Experimental Procedures

Optical rotations were measured on a JASCO P-1020 digital polarimeter (JASCO, Tokyo, Japan). The NMR spectra were recorded on a Bruker AVANCE 400 MHz spectrometer (Bruker, Ettlingen, Germany). HRESI-MS were obtained in the positive or negative ion mode with an AB SCIEX Trip TOFTM 5600+ (AB Sciex, Framingham, MA, USA). HPLC was carried out on a waters Breeze system (Waters, Milford, MA, USA) with an ODS column (Sunfire ODS, 4.6 × 250 mm, 5 μm,), Semipreparative HPLC was carried out on a waters Breeze system (Waters, Milford, MA, USA) with an ODS column (Sunfire ODS, 10 × 250 mm, 5 μm, 3 mL/min). MPLC was performed on a LC3000 series (Tong Heng Innovation Technology, Beijing, China) with a Flash C18 cartridge (50 μm, 40 g, YMC, Kyoto, Japan); all solvents were HPLC grade. Column chromatography was performed with silica (200–300 mesh, Qingdao Marine Chemical Inc, Qingdao, China) and Sephadex LH-20 (Amersham Biosciences Inc, Uppsala, Sweden), respectively. Thin layer chromatography (TLC) was carried out with glass precoated silica gel GF254 plates. The reagents for analysis were purchased from Xilong Scientific Co., Ltd. (Gongdong, China).

### 3.2. Fungus Material

The endophytic fungus DX-THL3, isolated from the healthy leaves of Dongxiang wild rice, was collected from a nature reserve in Dongxiang County, Jiangxi Province, China, in November 2013. This strain was deposited with the culture collection of the Key Laboratory of Protection and Utilization of Subtropical Plant Resources of Jiangxi Province, Jiangxi Normal University.

### 3.3. Identification of Strain DX-THL3

The endophytic fungus DX-THL3 was identified using both morphological characters and phylogenetic data. DX-THL3 was grown on the surface of potato dextrose agar (PDA) medium at 28 °C for 2 weeks, followed by identification based on the morphology of the fungal colony and the characteristics of the spores. Mycelia, conidia and pycnidia of the DX-THL3 strain were observed with a light microscope (BA300, Motic, Xiamen, China).

On the other hand, the DX-THL3 fungal strain was incubated in PDA for 7 days at 28 °C. Mycelia were scraped from the plate and ground to a powder under liquid nitrogen. Genomic DNA was then extracted using the Cetyltrimethylammonium Bromide (CTAB) method. The DNA was subjected to PCR amplification using the primers ITS1 (5′-TTCGTAGGTGAACCT GCGG-3′) and ITS4 (5′-TCCTCCGCTTATTGATAT GC-3′). The PCR reaction was performed in 50 µL of reaction mixture containing 25 µL of 2× Taq PCR Mastermix (Tiangen Biotech Beijing Co Ltd., Beijing, China), 2 µL of forward primer (10 µM), 2 mL of reverse primer (10 µM), 2 µL of template DNA and 19 µL of sterile double-distilled water. The PCR cycling protocol consisted of an initial denaturation at 94 °C for 3 min, 30 cycles of denaturation, annealing and elongation at 94 °C for 30 s, 60 °C for 30 s and 72 °C for 1 min. This protocol was followed by a final elongation step of 72 °C for 5 min. As a negative control, the template DNA was replaced by sterile double-distilled water. The PCR amplified products were analyzed using gel electrophoresis at approximately 500 bp–800 bp. The purification and sequencing of the PCR products was performed by Shanghai Invitrogen Company Ltd. (Shanghai, China). The corresponding sequence was analyzed through the basic local alignment search tool (BLAST). A neighbor-joining (NJ) phylogenetic tree was constructed in MEGA 6.0, using 1000 bootstrap replicates.

The ITS-rDNA of DX-THL3 was submitted to GenBank, and the accession number is KF558875.

### 3.4. Fermentation, Extraction and Metabolite Isolation

The endophytic fungus DX-THL3 was grown on PDA (200 g of potato, 20 g of glucose and 20 g of agar per liter of seawater) at 28 °C for 4 days, after which the agar was cut into pieces. The pieces (with a diameter of 0.5 cm) of mycelial agar were inoculated into 500 mL Erlenmeyer flasks (which each containing 200 mL of PDB medium) for 5 days on a rotary shaker (150 rpm). Then, the seed liquid was transferred into 500 Erlenmeyer flasks containing 150 mL of PDB medium each. The flasks were then incubated at 28 °C on a rotary shaker (180 rpm) for 15 days.

After 15 days of cultivation, the cultures (30 L) were filtered through cheesecloth to separate the mycelial mass from the aqueous layer. The filtrate was then extracted three times with an equal volume of ethyl acetate, followed by the removal of the ethyl acetate under vacuum to yield a crude extract (4.8 g). Then the crude extract was subjected to silica gel column chromatography (200 g), eluting successively with a petroleum ether/ethyl acetate gradient (99:1, 49:1, 19:1, 9:1, 4:1, 2:1, 1:1 and 0:1, *v*/*v*) and yielding six fractions (Fr.A–F). The bioactive fraction Fr.D (2.3 g) was chromatographed on Sephadex LH-20 (CH_2_Cl_2_-MeOH) to give three subfractions (Fr.D1-3). Subfraction Fr. D1 (94 mg) was purified via semipreparative HPLC using MeCN:H_2_O (60:40) with 0.1% formic acid as a mobile phase at a flow rate of 3 mL/min to furnish compound **2** (1.8 mg, *t_R_* 9.5 min). Subfractions Fr. D2 (1.1 g) was further purified via MPLC using MeCN:H_2_O (60:40) as a mobile phase at a flow rate of 10 mL/min to yield subfractions Fr. D2-1 and Fr. D2-2. Fr. D2-1(0.70 g) was further purified via semipreparative HPLC using MeCN:H_2_O (60:40) with 0.1% formic acid as a mobile phase at a flow rate of 3 mL/min to furnish compounds **5** (28 mg, *t_R_* 10.5 min), **4** (121 mg, *t_R_* 12.2 min) and **6** (11 mg, *t_R_* 14.6 min). Compounds **3** (8 mg, *t_R_* 16.8 min) and **7** (10.8 mg, *t_R_* 18.9 min) were purified using the same HPLC (MeCN:H_2_O (60:40) with 0.1% formic acid). The Fr.E (0.35 g) was chromatographed on Sephadex LH-20 (CH_2_Cl_2_-MeOH) to give four subfractions (Fr.E1-4). Subfraction Fr. E2 (34 mg) was further purified via semipreparative HPLC and eluted with a linear system (MeCN/H_2_O, 70:30) at a flow rate of 3 mL/min to furnish compound **1** (2.2 mg, *t_R_* 10.5 min).

### 3.5. Antibacterial Assay

The antibacterial activity in vitro against Gram-positive bacteria (*Staphylococcus aureus* (ATCC 29213) and *Bacillus subtilis* (ATCC 7508)) and Gram-negative bacteria (*Escherichia coli* (ATCC 25922) and *X. oryzae pv.oryzicola*) was evaluated via the 2-fold dilution assay in 96-well microtiter plates [23]. The bacterial strains were inoculated on Luria broth and incubated for 24 h at 37 °C; then, the cells were collected with normal saline and were diluted with broth to achieve 1.0 × 10^7^ mL^−1^. The cell broth was further diluted 10-fold before being adding into 96-well microtiter plates. 200 μL of mixtures was transferred into the first test well of each line in the 96-well while 100 μL was added in the other wells in the same line. Compounds were dissolved into DMSO and adjusted to 10 mg mL^−1^. Compounds were added into the first well at a concentration of 256 μg mL^−1^, then 100 μL was transferred to the second well until the final concentration of the twelfth well was 0.5 μg mL^−1^. Streptomycin sulphate and tobramycin were used as positive controls and DMSO was used as the negative control. The 96-well plates were incubated at 37 °C for 24 h, and the optical density (OD) was tested at 600 nm while using a microplate reader. The minimum inhibitory concentration (MIC) was defined as the minimal concentration of an antimicrobial compound that will inhibit the visible growth of a microorganism after 24 h incubation.

## 4. Conclusions

In summary, we discovered eight helvolic acid derivatives from the endophytic fungus *Sarocladium oryzae* of the Dongxiang wild rice (*Oryza rufipogon* Griff.) leaf, among which compounds **1**, **2** and **3a** are new. An antibacterial assay showed that compounds **4**–**7** exhibited somewhat antibacterial activities against *S. aureus*, which is consistent with previous results [19,21,22]. The new compound **2** is more potent against *S. aureus* than compounds **4** and **7**. Compound **5** showed the strongest amount of activity against *S. aureus*, which may serve for antibacterical agent use. Thus, for the antibacterial SAR of helvolic acid and its derivatives, it can be concluded that an α,β-unsaturated ketone unit in ring-A, an ortho–hydroxy carbonyl or an α-orientation hydroxyl substitution at C-7 in ring-B contribute to antibacterial activity. Meanwhile, the presence of a C-21/C-16 lactone or a C-21/C-24 lactone will significantly reduce antibacterial activity.

## Figures and Tables

**Table 1:** ^1^H (400 MHz) and ^13^C NMR (100 MHz) data of compounds **1**–**3**.

Position	1 ^a^		2 ^b^		3a ^b^		3b ^b^	
	δ_C_	δ_H_ (*J* in Hz)	δ_C_	δ_H_ (*J* in Hz)	δ_C_	δ_H_ (*J* in Hz)	δ_C_	δ_H_ (*J* in Hz)
1	157.8, CH	7.30 d (10.0)	161.3, CH	7.42 d (10.2)	33.8, CH_2_	2.02 m, 1.66 m	160.2, CH	7.50 (d 10.0)
2	127.7, CH	5.85 d (10.0)	127.9, CH	5.80 d (10.2)	37.4, CH_2_	2.50 m	128.2, CH	5.83 (d 10.0)
3	202.1, C		204.3, C		215.7, C		203.7, C	
4	40.0, CH	3.02 dq (12.5, 6.6)	44.0, CH	2.38 m	43.2, CH	2.59 m	41.5, CH	2.83 m
5	47.2, CH	2.22 br d (12.5)	43.4, CH	2.41 dd (11.4, 5.7)	46.4, CH	2.22 m	48.3, CH	2.41 m
6	73.5, CH	4.03 br s	34.4, CH_2_	1.92 m	75.0, CH^c^	5.19 br s	74.8, CH^c^	5.18 br s
				1.65 m				
7	213.2, C		70.6, CH	4.04 dd (8.4, 4.1)	211.3, C		211.9, C	
8	52.3, C		46.2, C		54.2, C		54.0, C	
9	41.6, CH	2.67 dd (13.2, 2.5)	47.1, CH	1.66 m	43.1, CH	2.65 m	42.6, CH	2.65 m
10	38.2, C		40.2, C		36.2, C		39.5, C	
11	24.3, CH_2_	1.96 m	26.3, CH_2_	2.03 m	23.7, CH_2_		24.8, CH_2_	
		1.57 m		1.54 m				
12	26.6, CH_2_	2.47 m	22.9, CH_2_	2.14 m	27.3, CH_2_	2.39 m	27.3, CH_2_	2.39 m
		1.82 m		1.76 m		1.74 m		1.74 m
13	51.6, CH	2.62 br d (12.3)	44.5, CH	2.99 dd (12.5, 2.5)	49.8, CH	2.70 m	49.6, CH	2.70 m
14	46.8, C		56.3, C		48.1, C		47.9, C	
15	41.1, CH_2_	2.12 m	38.2, CH_2_	2.48 dd (15.2, 11.1)	41.9, CH_2_	2.33 m	41.7, CH_2_	2.33 m
		2.12 m		1.32 dd (15.2, 4.7)		1.75 m		1.75 m
16	73.8, CH	5.98 br d (6.8)	84.1, CH	4.99 dd (11.1, 4.7)	75.1, CH^c^	5.79 (d 8.4)	75.1, CH^c^	5.79 (d 8.4)
17	155.0, C		172.3, C		145.7, C		145.7, C	
18	18.9, CH_3_	1.05 s	20.7, CH_3_	0.93 s	18.5, CH_3_	0.91 s	18.4, CH_3_	0.93 s
19	28.3, CH_3_	1.52 s	25.8, CH_3_	1.14 s	24.1, CH_3_	1.31 s	27.8, CH_3_	1.49 s
20	125.1, C		124.4, C		133.3, C		133.2, C	
21	164.8, C		178.8, C		174.2, C		174.1, C	
22	24.7, CH_2_	3.11 br d (15.2)	24.9, CH_2_	2.36 m	25.6, CH_2_	1.48 m	25.6, CH_2_	1.48 m
		2.44 m						
23	24.0, CH_2_	2.06 m	28.3, CH_2_	2.27 m	29.8, CH_2_	2.58 m	29.8, CH_2_	2.58 m
		1.74 m				2.34 m		2.34 m
24	85.3, CH	4.09 dd (11.0, 3.6)	124.4, CH	5.14 m	44.2, CH_2_	1.48 m	44.2, CH_2_	1.48 m
25	71.8, C		133.8, C		71.2, C		71.2, C	
26	25.6, CH_3_	1.27 s	17.8, CH_3_	1.60 s	29.3, CH_3_^d^	1.17 s	29.2, CH_3_^d^	1.17 s
27	24.2, CH_3_	1.22 s	25.8, CH_3_	1.68 s	29.1, CH_3_^d^	1.18 s	29.1, CH_3_^d^	1.18 s
28	12.5, CH_3_	1.23 d (7.1)	12.8, CH_3_	1.10 d (6.1)	13.4, CH_3_	1.13 (d 6.8)	13.1, CH_3_	1.24 (d 6.8)
30	17.5, CH_3_	1.13 s	14.6, CH_3_	1.12 s	17.7, CH_3_	1.36 s	18.7, CH_3_	1.21 s
6-OAC					20.6, CH_3_^e^	1.97 s	20.6, CH_3_^e^	1.97 s
					172.4, C		172.4, C	
16-OAC	169.9, C				20.7, CH_3_^e^	2.10 s	20.7, CH_3_^e^	2.12 s
	20.8, CH_3_	2.01 s			170.9, C		170.9, C	

Recorded at 400 or 100 MHz for ^1^H and ^13^C, a in CDCl_3_, b in CD_3_OD; c, d and e may be interchangeable.

**Table 2:** ^1^H (400 MHz) and ^13^C NMR (100 MHz) data of compounds 4–7.

Position	4 ^a^		5 ^a^		6 ^a^		7 ^b^	
	δ_C_	δ_H_ (*J* in Hz)	δ_C_	δ_H_ (*J* in Hz)	δ_C_	δ_H_ (*J* in Hz)	δ_C_	δ_H_ (*J* in Hz)
1	157.2, CH	7.31, d (10.1)	158.4, CH	7.33, d (10.0)	157.6, CH	7.34, d (10.0)	33.8, CH_2_	
2	127.8, CH	5.87, d (10.1)	127.5, CH	5.85, d (10.0)	128.4, CH	5.85, d (10.0)	37.4, CH_2_	
3	201.4, C		202.4, C		201.5, C		215.7, C	
4	40.4, CH	2.78, m	39.9, CH	3.05, m	41.9, CH	2.56, m	42.6, CH	
5	47.2, CH	2.27, d (11.3)	47.1, CH	2.15, d (12.4)	43.7, CH	2.06, overlapped	46.4, CH	2.22, d (12.3)
6	73.8, CH	5.24, s	73.9, CH	3.99, s	39.8, CH_2_	2.37, m	75.1, CH	5.19, s
						2.24, m		
7	208.8, C		216.0, C		216.1, C		211.3, C	
8	52.6, C		52.4, C		52.15, C	2.06, overlapped	54.2, C	
9	41.7, CH	2.62, dd (13.3, 6.7)	41.3, CH	2.70, br d(12.8)	43.3, CH		43.2, CH	
10	38.1, C		38.3, C		38.5, C		36.2, C	
11	23.9, CH_2_	1.97, m	24.0, CH_2_	1.97, m	24.2, CH_2_	1.93, m	23.7, CH_2_	
		1.58, m		1.55, m		1.51 m		
12	25.9, CH_2_	2.43, m	25.9, CH_2_	2.40, m	25.9, CH_2_	2.39, m	27.4, CH_2_	
		1.83, m		1.82, m		1.76, m		
13	49.4, CH	2.60, dd (13.3, 6.7)	49.6, CH	2.57, br d (11.7)	49.3, CH	2.53, overlapped	49.7, CH	2.67, br d (11.6)
14	46.6, C		46.5, C		46.8, C		48.1, C	
15	40.6, CH_2_	2.25, dd (13.3, 6.7)	40.8, CH	2.24, m	40.6, CH_2_	2.22, m	41.7, CH_2_	2.32,dd(13.3,6.7)
		1.92, d (15.0)		1.83, m		1.91, m		1.74, d (14.7)
16	73.4, CH	5.88, d (8.3)	73.8, CH	5.85, d (10.0)	73.6, CH	5.87, br d (9.4)	74.8,CH	5.78, d (8.3)
17	147.9, C		148.5, C		147.8, C		146.2, C	
18	17.9, CH_3_	0.93, s	18.2, CH_3_	0.95, s	17.4, CH_3_	0.90, s	17.8, CH_3_	0.91, s
19	27.5, CH_3_	1.45, s	28.1, CH_3_	1.55, s	25.5, CH_3_	1.30, s	24.1, CH_3_	1.31, s
20	130.2, C		130.4, C		130.3, C		132.9, C	
21	173.5, C		174.2, C		174.6, C		174.0, C	
22	28.6, CH_2_	2.48, m	28.4, CH_2_	2.48, m	28.5, CH_2_	2.48, m	29.6, CH_2_	
								
23	28.3, CH_2_	2.10, m	28.4, CH_2_	2.06, m	28.3, CH_2_	2.08, m	29.1, CH_2_	
				1.62, m				
24	122.7, CH	5.11, m	122.8, CH	5.10, (t like)	122.8, CH	5.10, m	124.3, CH	5.14, t (7.0)
25	133.0, C		132.9, C		132.9, C		133.4, C	
26	25.7, CH_3_	1.70, s	25.7, CH_3_	1.69, s	25.7, CH_3_	1.69, s	25.9, CH_3_	1.68, s
27	17.8, CH_3_	1.61, s	17.7, CH_3_	1.60, s	17.8, CH_3_	1.60, s	17.7, CH_3_	1.62, s
28	13.1, CH_3_	1.28, d (6.8)	12.4, CH_3_	1.21, d (6.6)	13.0, CH_3_	1.15, d (6.6)	13.4, CH_3_	1.12, d (6.7)
30	18.3, CH_3_	1.18, s	17.9, CH_3_	1.13, s	17.7, CH_3_	1.11, s	18.5, CH_3_	1.35, s
6-OAC	168.9, C						172.4, C	
	20.8, CH_3_	2.12, s					20.6, CH_3_	1.96, s
16-OAC	170.1, C		170.9, C		170.5, C		170.9, C	
	20.5, CH_3_	1.95, s	20.4, CH_3_	1.97, s	20.5, CH_3_	1.97, s	20.7, CH_3_	2.10, s

Recorded at 400 or 100 MHz for ^1^H and ^13^C, a in CDCl_3_, b in CD_3_OD.

**Table 3:** The inhibitory effects of compounds **1**–**7** on bacteria.

Compounds	MIC (μg/mL)			
	*S. aureus* (ATCC 29213)	*B. subtilis*	*E. coli* (ATCC 25922)	*X. oryzae pv.oryzicola*
**1**	64	>128	>128	>128
**2**	4	>128	64	>128
**4**	8	>128	>128	>128
**5**	1	64	64	>128
**6**	4	>128	>128	>128
**7**	16	>128	64	>128
Streptomycin sulphate	NT	NT	8	64
tobramycin	1	64	NT	NT

NT: not test; MIC: minimum inhibitory concentration.

## Data Availability

Data is contained within the article or supplementary material.

## Supplementary Materials

Supporting information for this article is available online, Figures S1–S34 associated with the morphological characteristics and phylogenetic tree of strain DX-THL3, HR-ESI-MS and the 1D and 2D NMR spectra data for compounds **1**–**7**.

## Sample Availability

Samples of the compounds **1**-**7** are available from the authors.
