# Supplementary material for: New Helvolic Acid Derivatives with Antibacterial Activities from Sarocladium oryzae DX-THL3, an Endophytic Fungus from Dongxiang Wild Rice (Oryza rufipogon Griff.)
Source: Molecules. 2021 Mar 24;26(7):1828. doi: 10.3390/molecules26071828 (PMC8037696; doi:10.3390/molecules26071828)
Supplement: Supplementary file 1 [file molecules-26-01828-s001.pdf]

SUPPLEMENTARY MATERIAL

Three new Helvolic acid derivatives from *Sarocladium oryzae* DX-THL3, an endophytic fungus from DongXiang wild Rice (*Oryza rufipogon* Griff.)

Zhi-Bin Zhang[1], Si-Yao Du[1], Bo Ji[3], Chang-Jiu Ji*[,2], Yi-Wen Xiao[1,3], Ri-ming Yan[1], and Du Zhu *[,1,3]

[1] Key Laboratory of Protection and Utilization of Subtropical Plant Resources of Jiangxi Province, College of Life Science, Jiangxi Normal University, Nanchang, 330022,China; zzbbio@jxnu.edu.cn(Z.-B. Z.); 1294121506@qq.com(S.-Y. D.); rimimgyan@163.com(R.-M. Y.); zhudu12@163.com(D. Z.)

[2] College of Chemistry and Biological Engineering, Yichun University, Jiangxi, 336000,China; jichjiu999@jxycu.edu.cn(C.-J. J.)

[3] College of Life Science, Jiangxi Science and Technology Normal University, Nanchang,330013, China; 1152858687@qq.com (Y.-W. X.); 1072820639@qq.com(B. J.)

* Correspondence: zhudu12@163.com;Tel.:+86-795-88121934(D. Z.).
jichjiu999@jxycu.edu.cn;Tel.: +86-15279850892(C.-J. J.).

Figure list: Morphological characters and phylogenetic tree of Strain DX-THL3; HR-ESI-MS, 1D and 2D NMR spectra data for compounds 1-7

Figure S1. Morphological characters and phylogenetic tree of Strain DX-THL3
Figure S2. ^{1}H NMR(400MZ) spectrum of compound **1** in CD_3OD
Figure S3. ^{13}C NMR(100MZ) and DEPT spectrum of compound **1** in CD_3OD
Figure S4. ^{1}H-^{1}H COSY spectrum of compound **1** in CD_3OD
Figure S5. HSQC spectrum of compound **1** in CD_3OD
Figure S6. HMBC spectrum of p compound **1** in CD_3OD
Figure S7. NOESY spectrum of compound **1** in CD_3OD
Figure S8. HRESIMS spectrum of compound **1**
Figure S9. ^{1}H NMR(400MZ) spectrum of compound **2** in $CDCl_3$
Figure S10. ^{13}C NMR(100MZ) and DEPT spectrum of compound **2** in $CDCl_3$
Figure S11. ^{1}H-^{1}H COSY spectrum of compound **2** in $CDCl_3$
Figure S12. HSQC spectrum of compound **2** in $CDCl_3$
Figure S13. HMBC spectrum of p compound **2** in $CDCl_3$
Figure S14. NOESY spectrum of compound **2** in $CDCl_3$
Figure S15. HRESIMS spectrum of compound **2**
Figure S16. ^{1}H NMR(400MZ) spectrum of compounds **3a** and **3b** in CD_3OD
Figure S16. Expansion ^{1}H NMR(400MZ) spectrum of compounds **3a** and **3b** in CD_3OD
Figure S17. ^{13}C NMR and DEPT spectrum of compounds **3a** and **3b** in CD_3OD
Figure S17. Expansion ^{13}C NMR and DEPT spectrum of compounds **3a** and **3b** in CD_3OD
Figure S17. Expansion ^{13}C NMR and DEPT spectrum of compounds **3a** and **3b** in CD_3OD
Figure S17. Expansion ^{13}C NMR and DEPT spectrum of compounds **3a** and **3b** in CD_3OD
Figure S18. ^{1}H-^{1}H COSY spectrum of compounds **3a** and **3b** in CD_3OD
Figure S19. HSQC spectrum of compounds **3a** and **3b** in CD_3OD
Figure S20. HMBC spectrum of compounds **3a** and **3b** in CD_3OD
Figure S21. NOESY spectrum of compounds **3a** and **3b** in CD_3OD
Figure S22. HRESIMS spectrum of compounds **3a** and **3b**
Figure S23. ^{1}H NMR(400MZ) spectrum of compound **5** in $CDCl_3$
Figure S24. ^{13}C NMR(100MZ) and DEPT spectrum of compound **5** in $CDCl_3$
Figure S25. ^{1}H-^{1}H COSY spectrum of compound **5** in $CDCl_3$
Figure S26. HSQC spectrum of compound **5** in $CDCl_3$
Figure S27. HMBC spectrum of compound **5** in $CDCl_3$
Figure S28. ^{1}H NMR(400MZ) spectrum of compound **6** in $CDCl_3$
Figure S29. ^{13}C NMR(100MZ) and DEPT spectrum of compound **6** in $CDCl_3$
Figure S30. ^{1}H-^{1}H COSY spectrum of compound **6** in $CDCl_3$
Figure S31. HSQC spectrum of compound **6** in $CDCl_3$
Figure S32. HMBC spectrum of p compound **6** in $CDCl_3$
Figure S33. ^{1}H NMR(400MZ) spectrum of compound **7** in CD_3OD
Figure S34. ^{13}C NMR(100MZ) and DEPT spectrum of compound **7** in CD_3OD

Figure S1. Morphological characters and phylogenetic tree of Strain DX-THL3

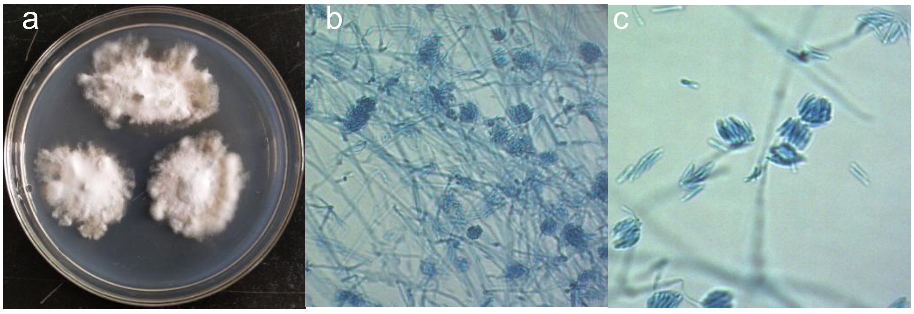

Colony and conidia of DX-THL3 on PDA medium

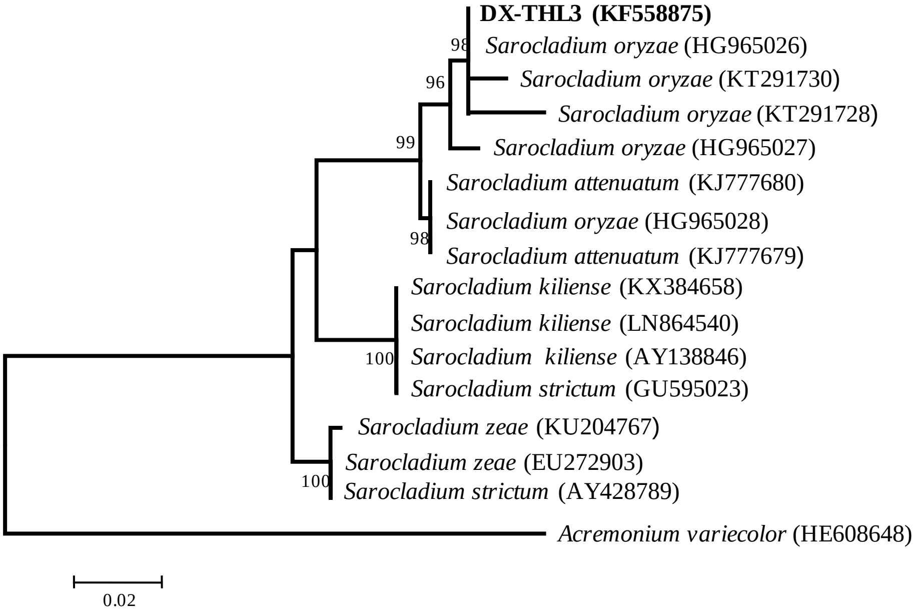

Phylogenetic NJ tree inferred from ITS rDNA sequences (Bar represents 0.02 substitutions per site)

Figure S2. ^{1}H NMR spectrum of compound **1** in $CDCl_3$

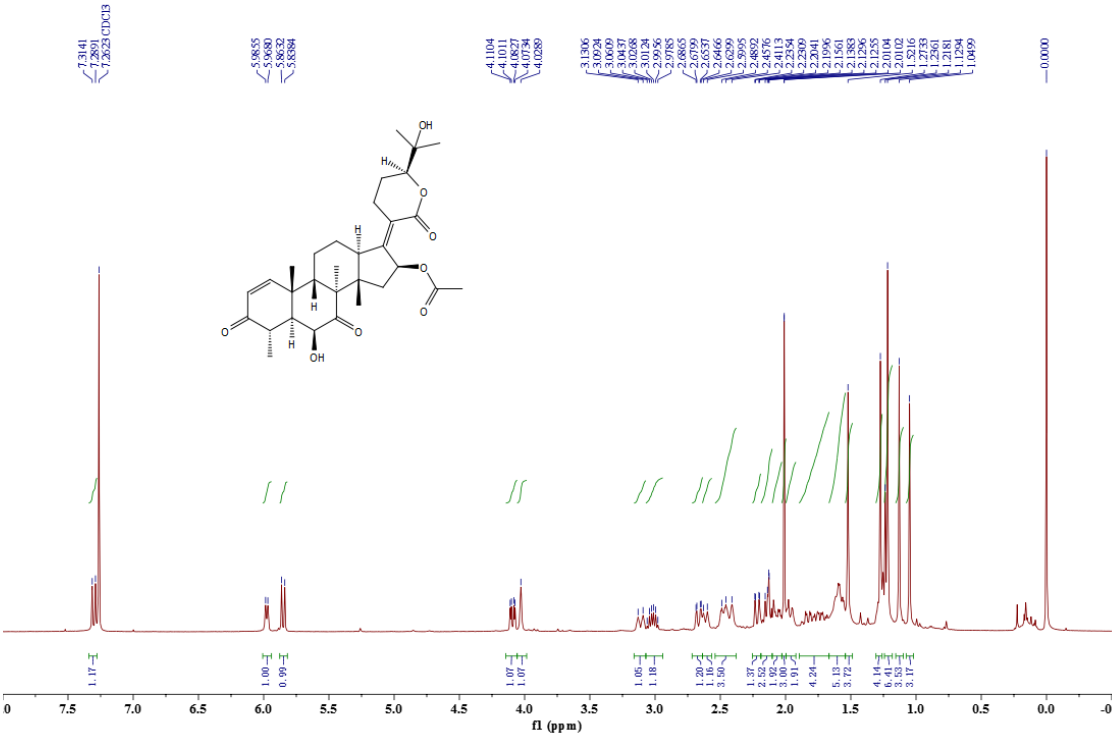

Figure S3. ^{13}C NMR and DEPT spectrum of compound **1** in $CDCl_3$

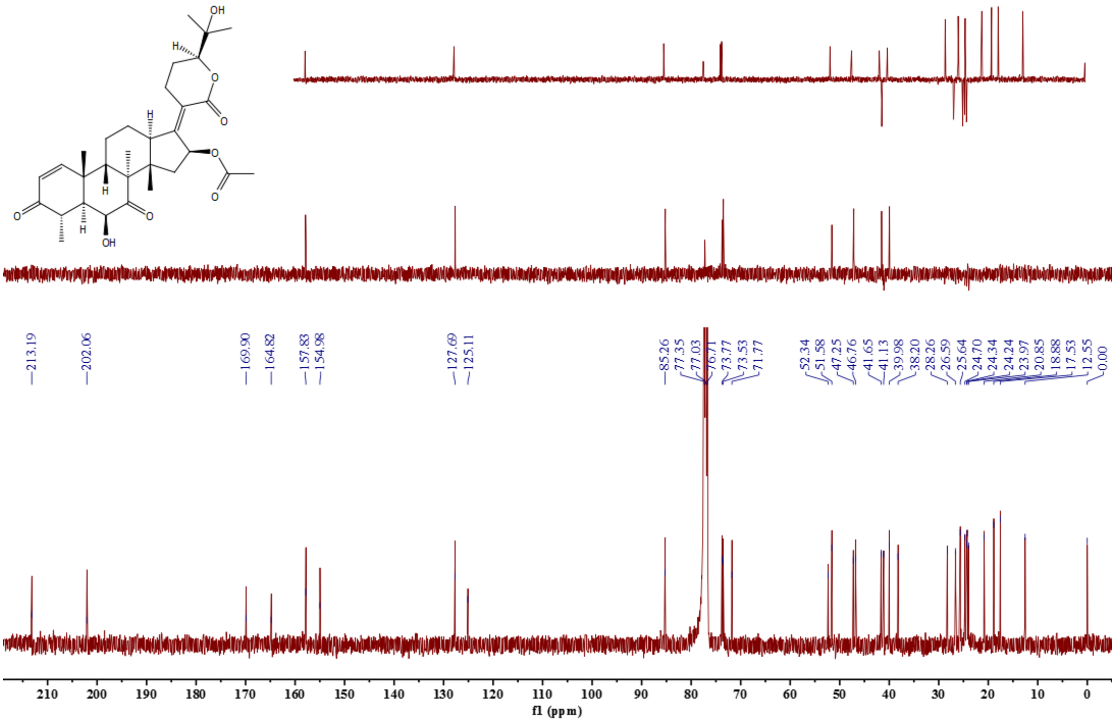

Figure S4. ^{1}H-^{1}H COSY spectrum of compound **1** in $CDCl_3$

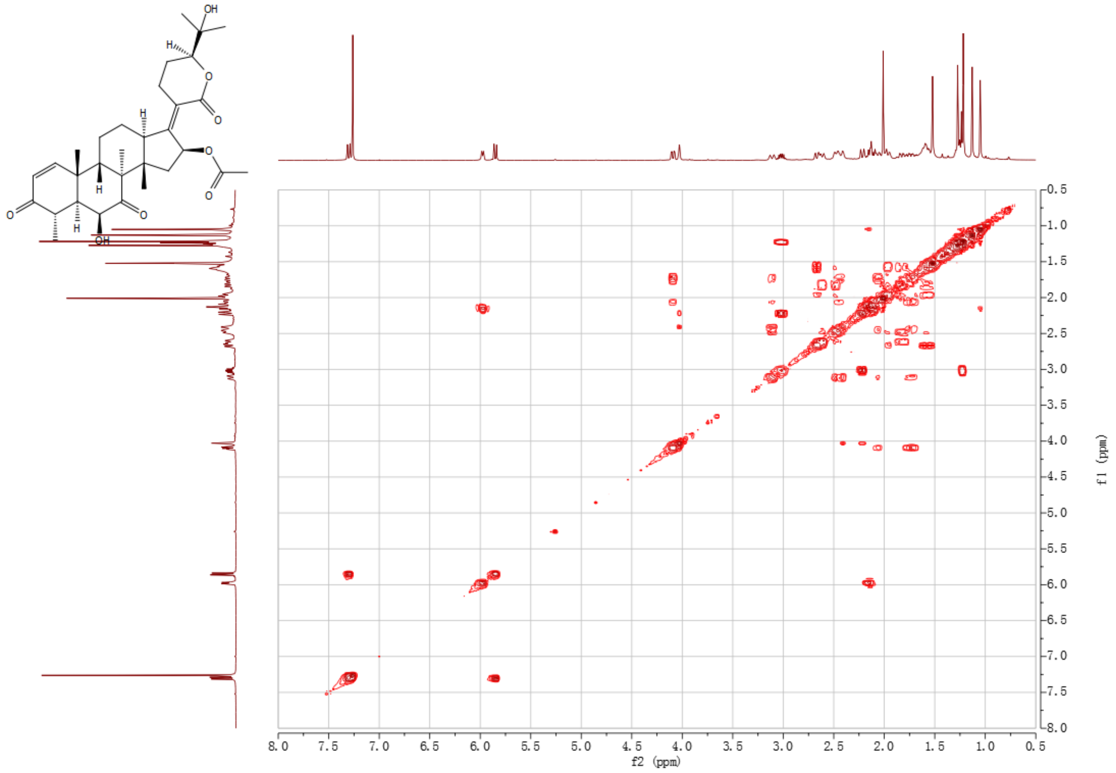

Figure S5. HSQC spectrum of compound **1** in $CDCl_3$

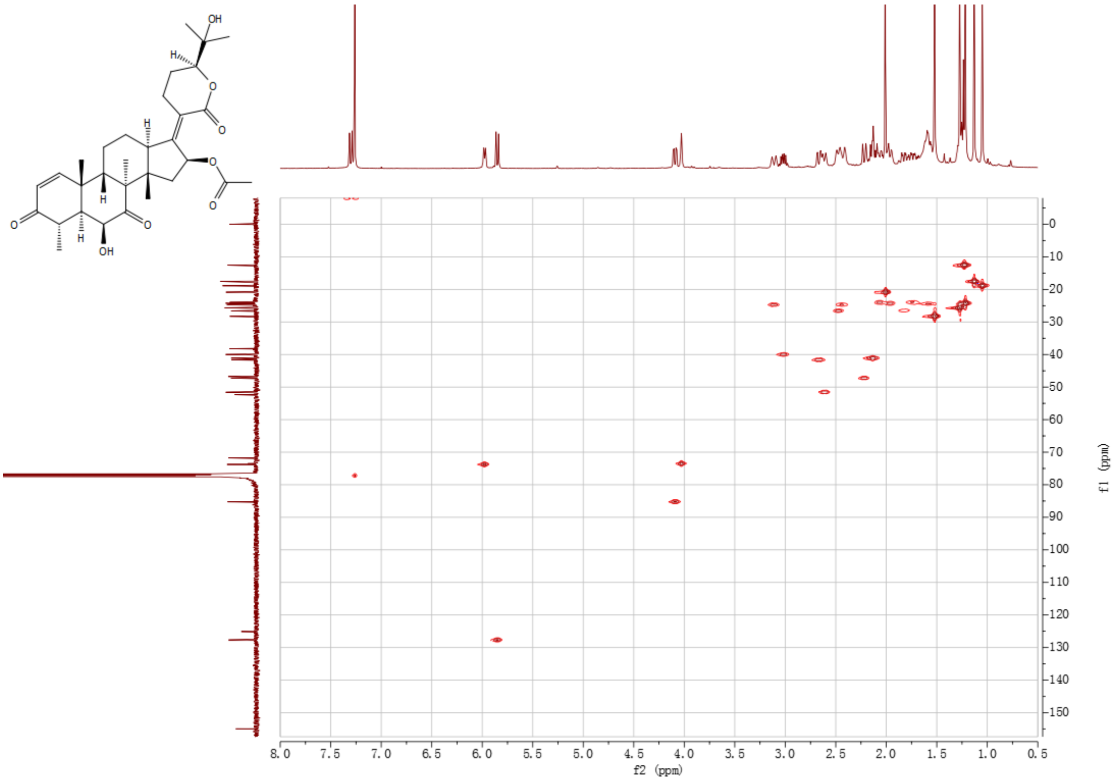

Figure S6. HMBC spectrum of compound **1** in $CDCl_3$

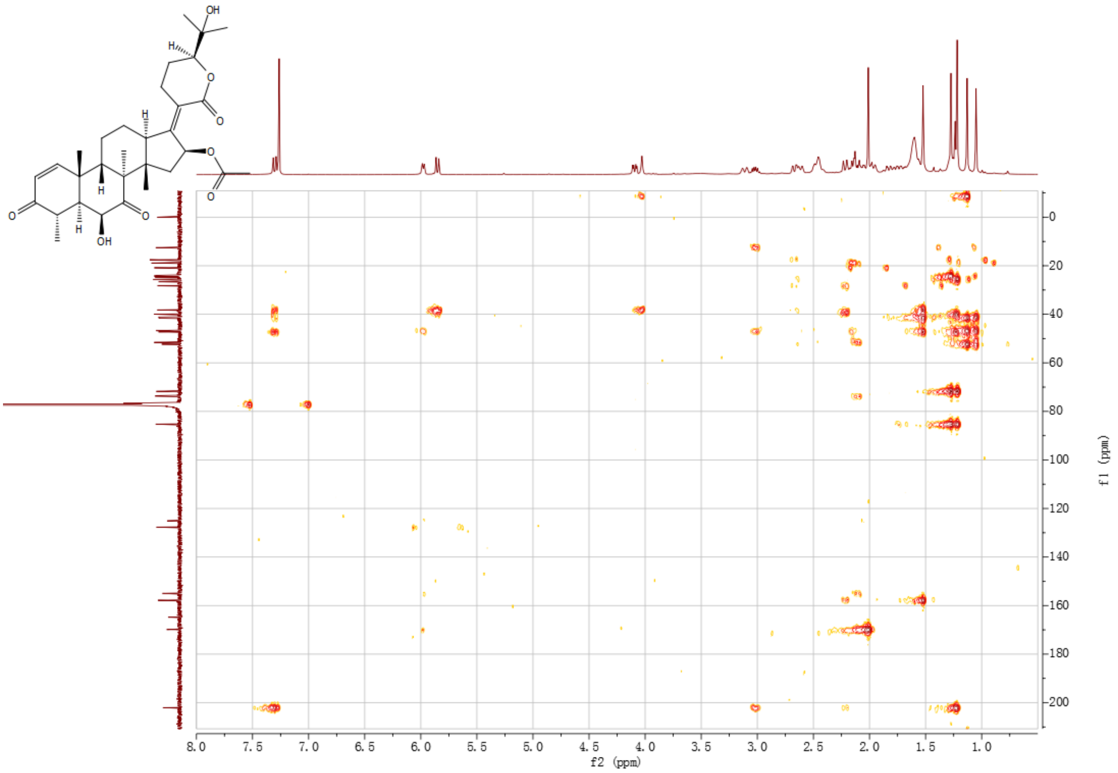

Figure S7. ROESY spectrum of compound **1** in $CDCl_3$

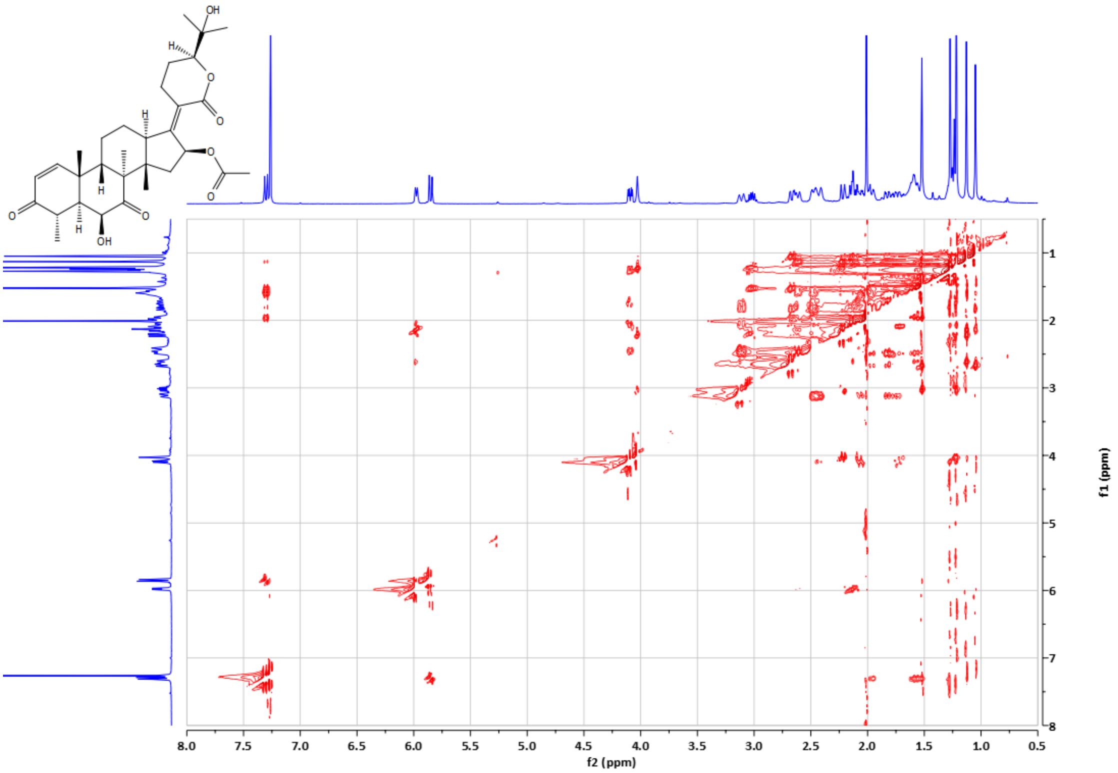

Figure S8. HRESIMS spectrum of compound **1**

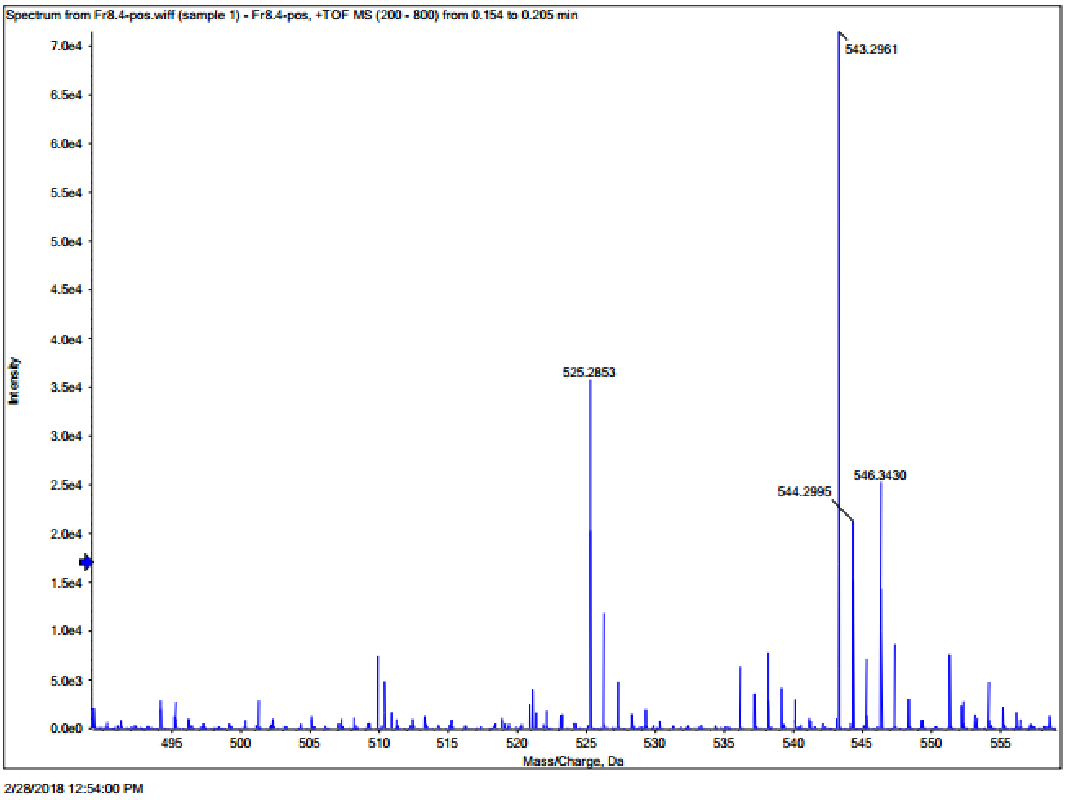

Figure S9. ^{1}H NMR spectrum of compound **2** in CD_3OD

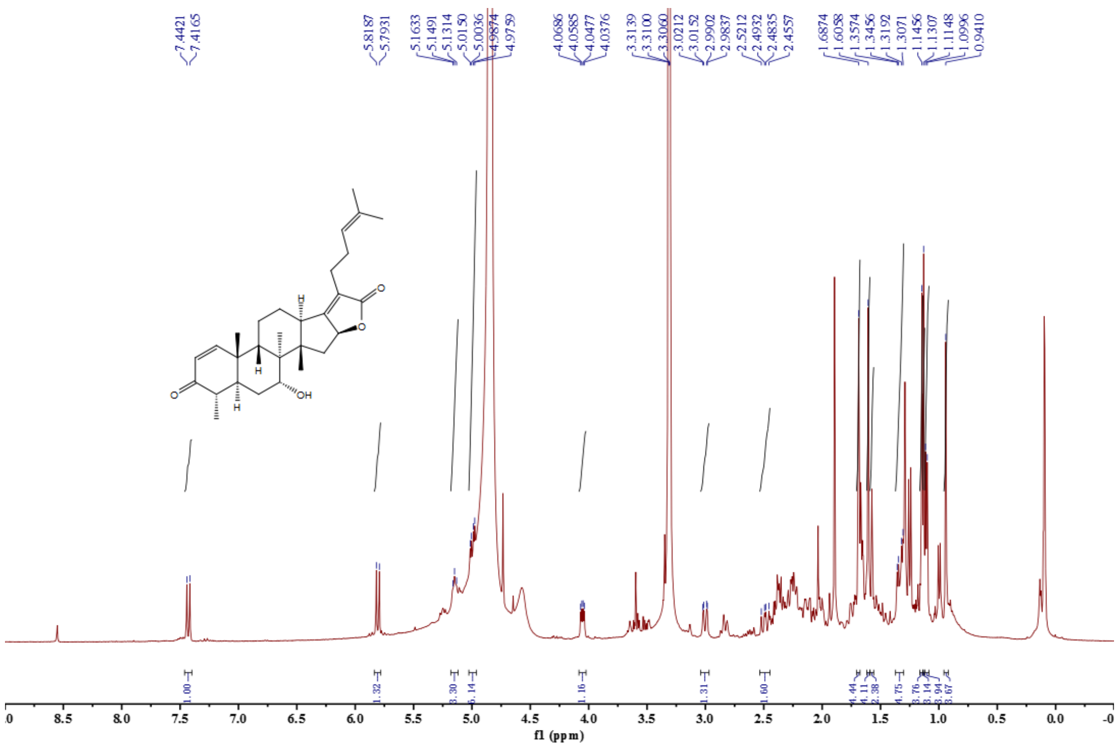

Figure S10. ^{13}C NMR and DEPT spectrum of compound **2** in CD_3OD

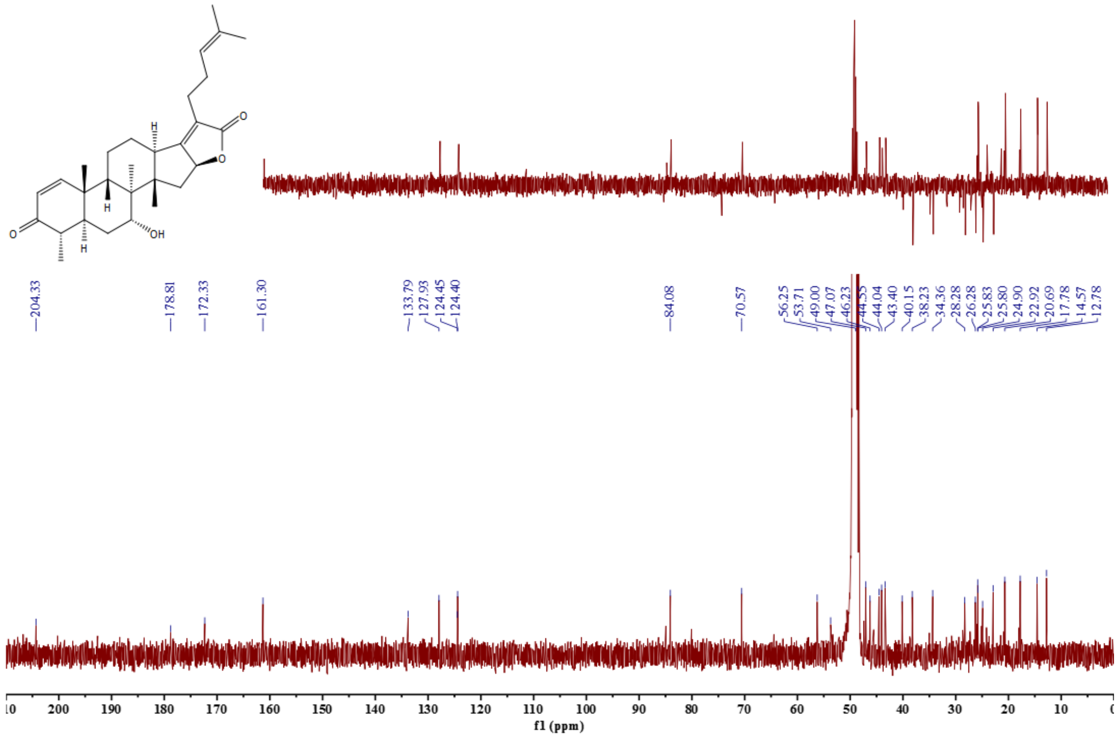

Figure S11. ^{1}H-^{1}H COSY spectrum of compound **2** in CD_3OD

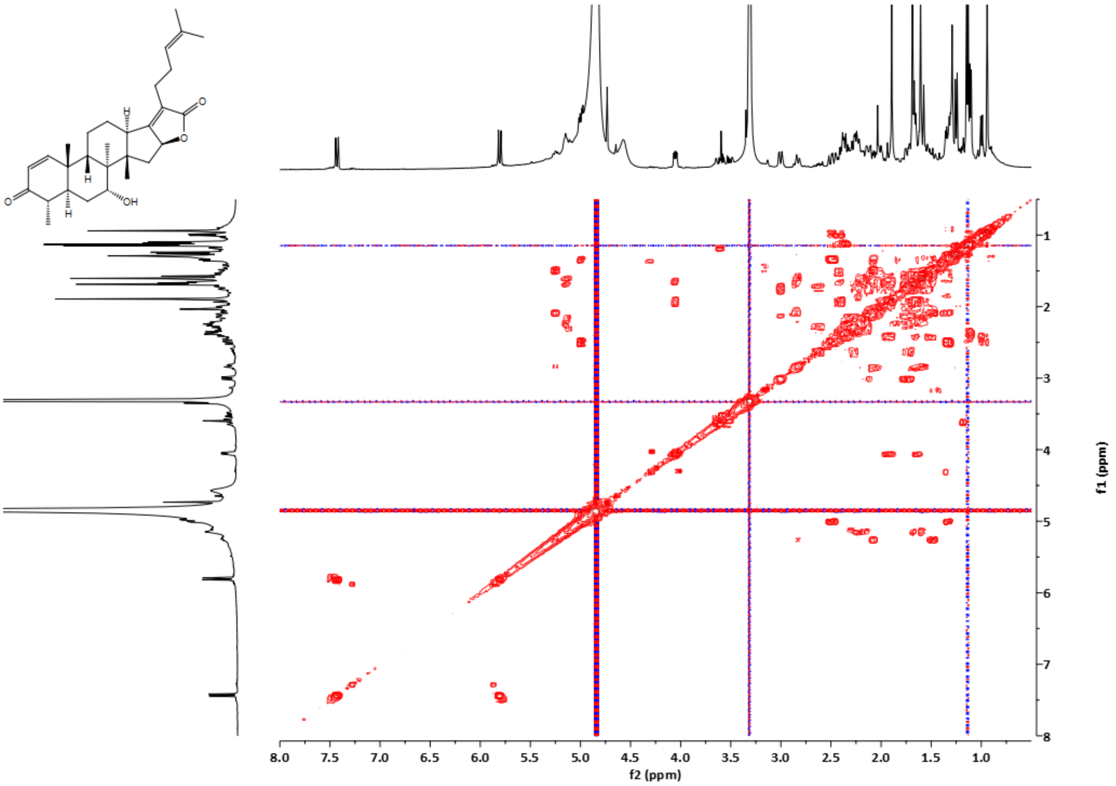

Figure S12. HSQC spectrum of compound **2** in CD_3OD

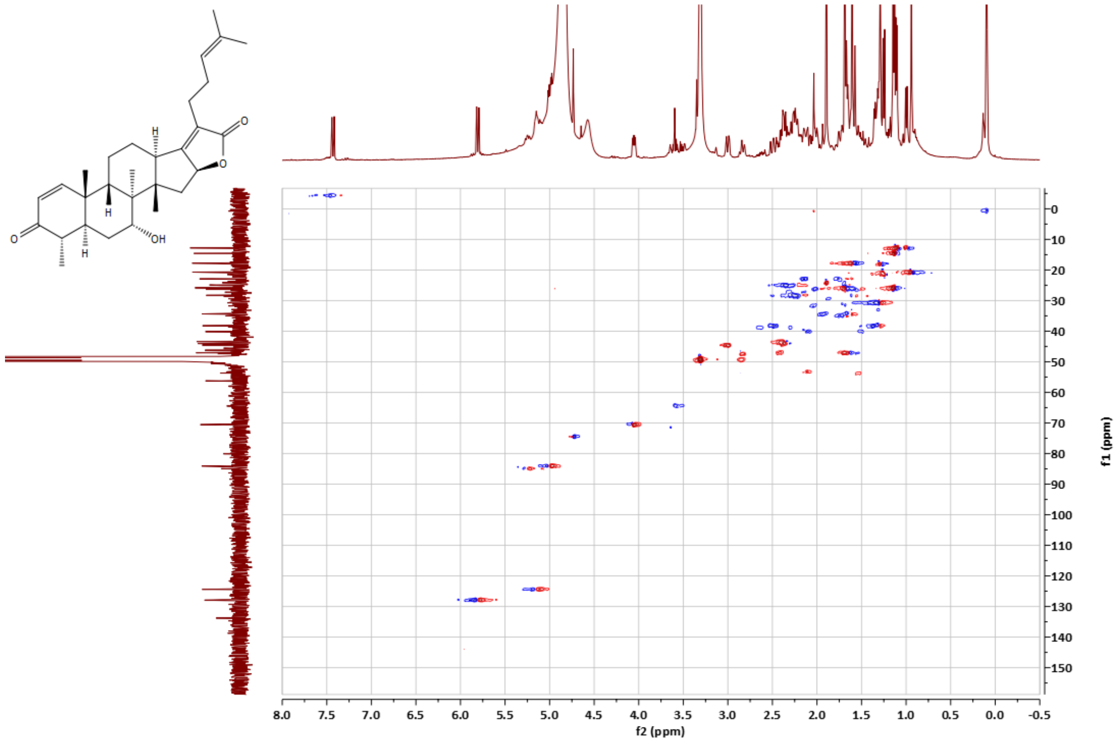

Figure S13. HMBC spectrum of compound **2** in CD_3OD

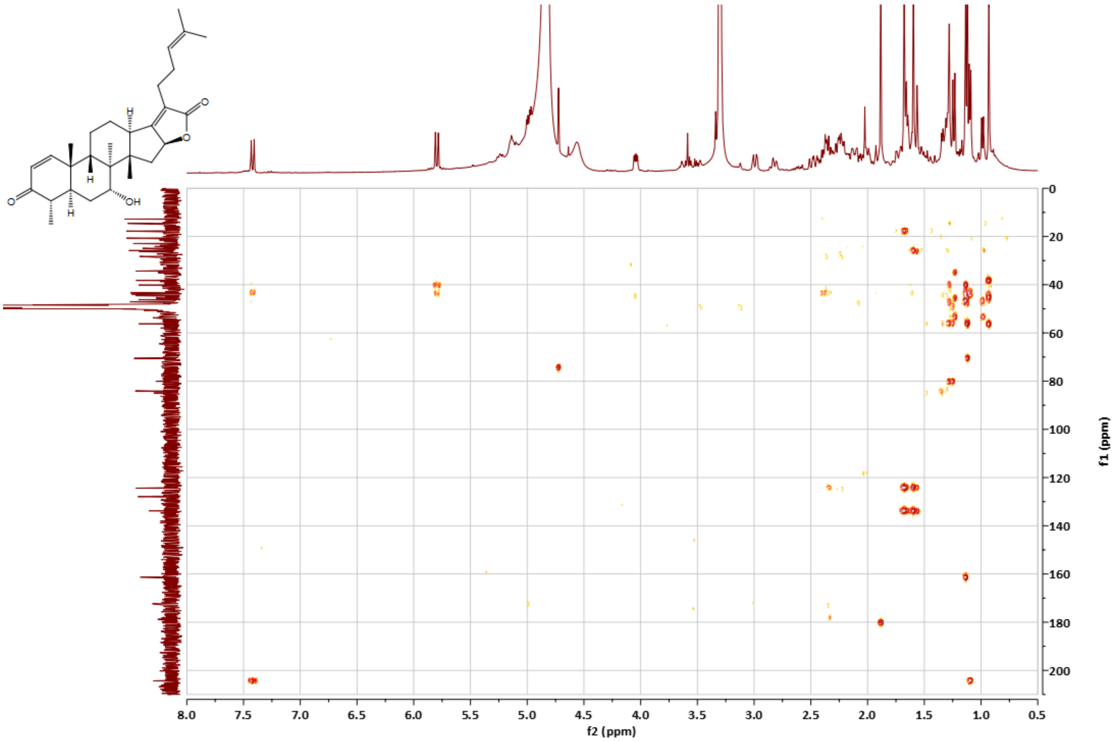

Figure S14. ROESY spectrum of compound **2** in CD_3OD

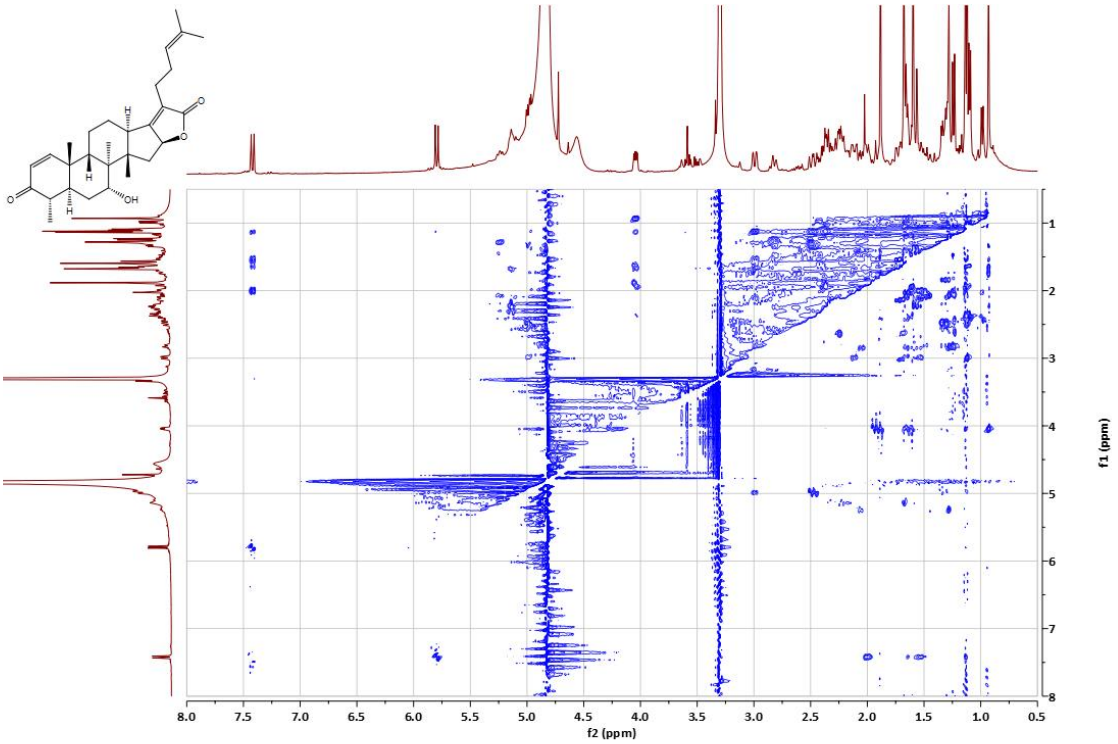

Figure S15. HRESIMS spectrum of compound **2**

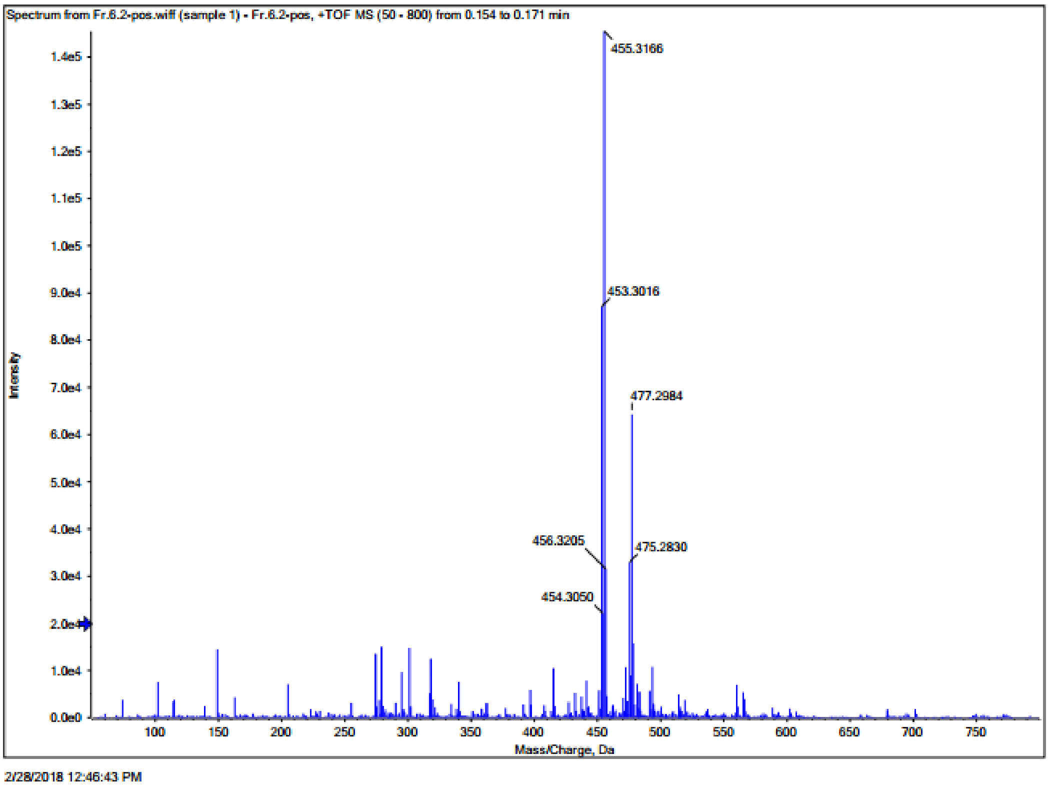

Figure S16. ^{1}H NMR spectrum of compound **3a** and **3b** in CD_3OD

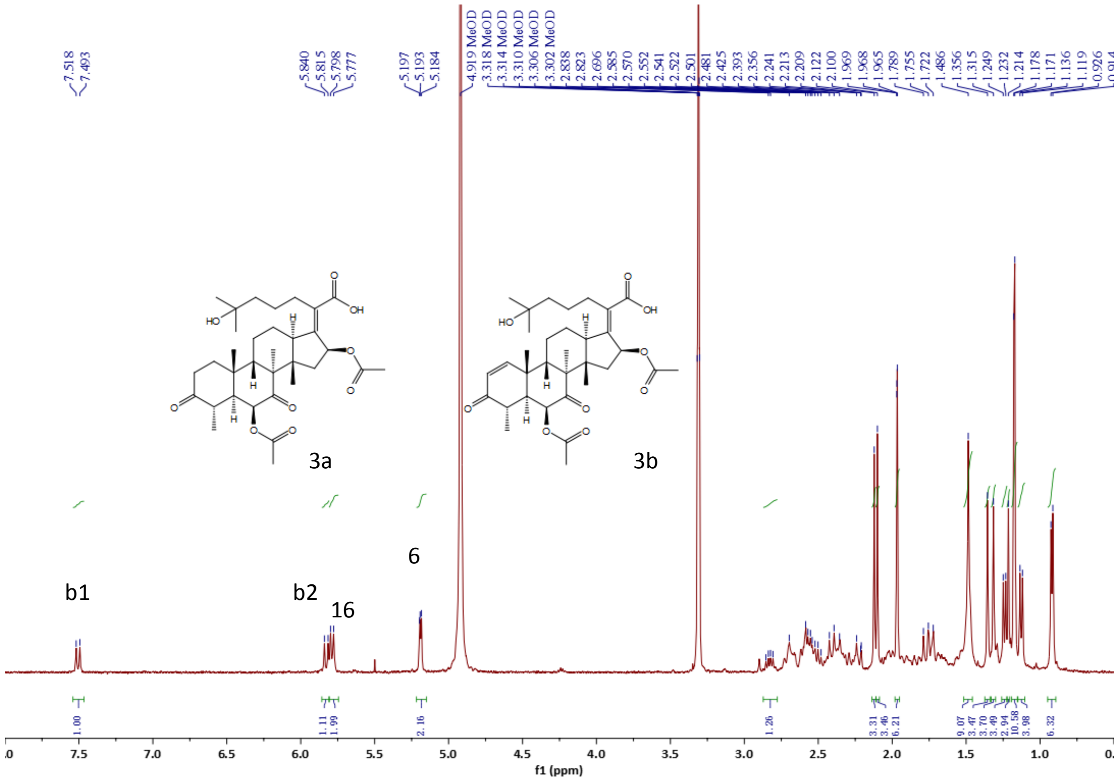

Figure S16. Expansion ^{1}H NMR spectrum of compound **3a** and **3b** in CD_3OD

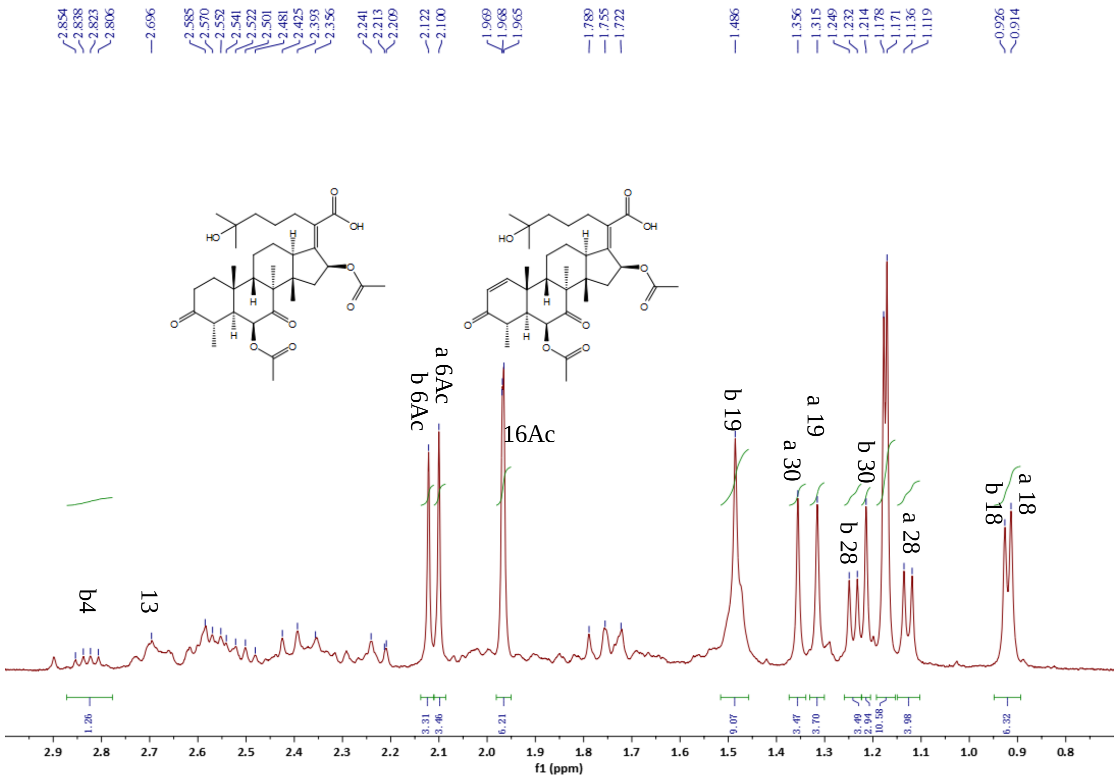

Figure S17. ^{13}C NMR and DEPT spectrum of compound **3a** and **3b** in CD_3OD

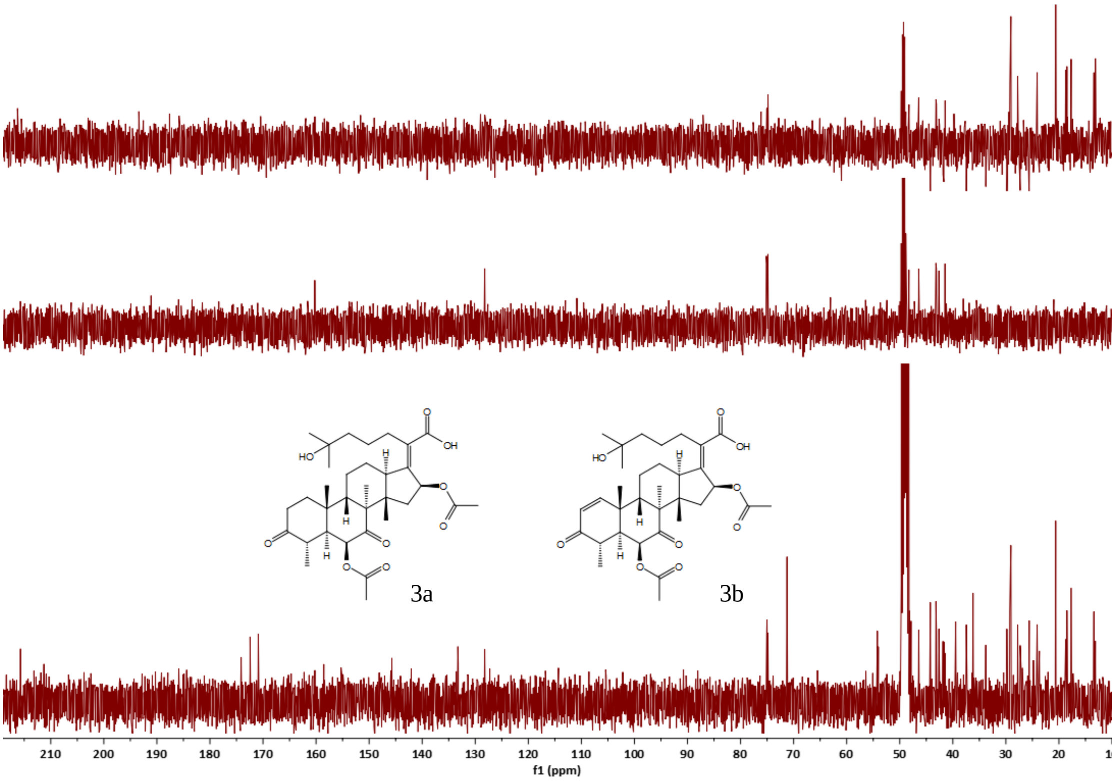

Figure S17. Expansion ^{13}C NMR and DEPT spectrum of compound **3a** and **3b** in CD_3OD

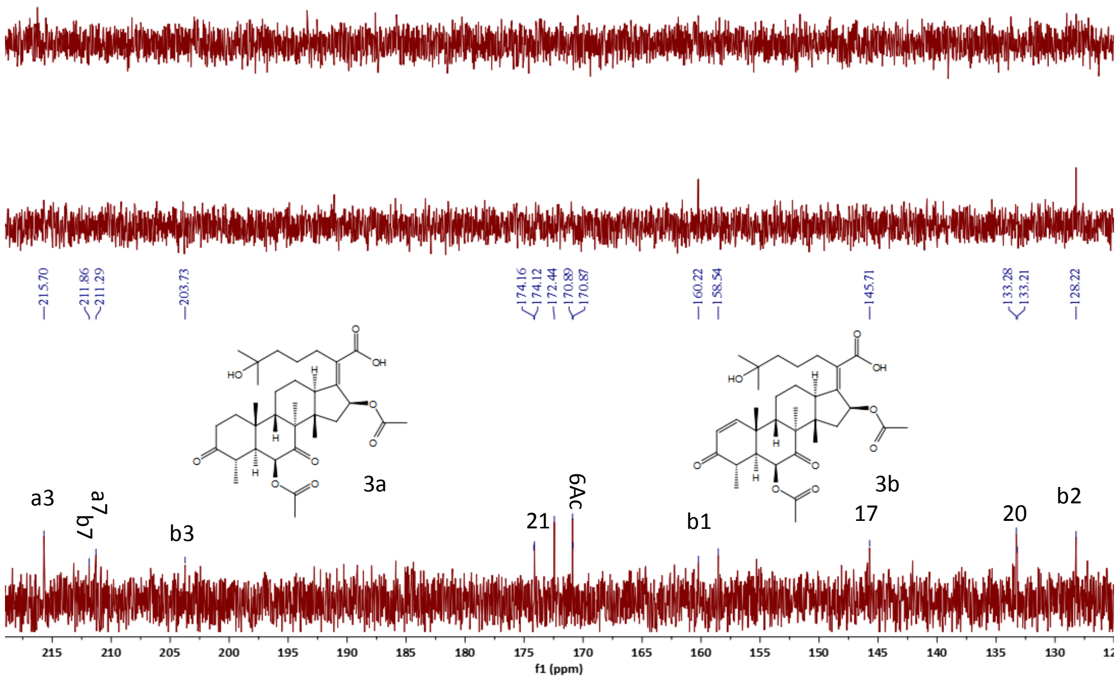

Figure S17. Expansion ^{13}C NMR and DEPT spectrum of compound **3a** and **3b** in CD_3OD

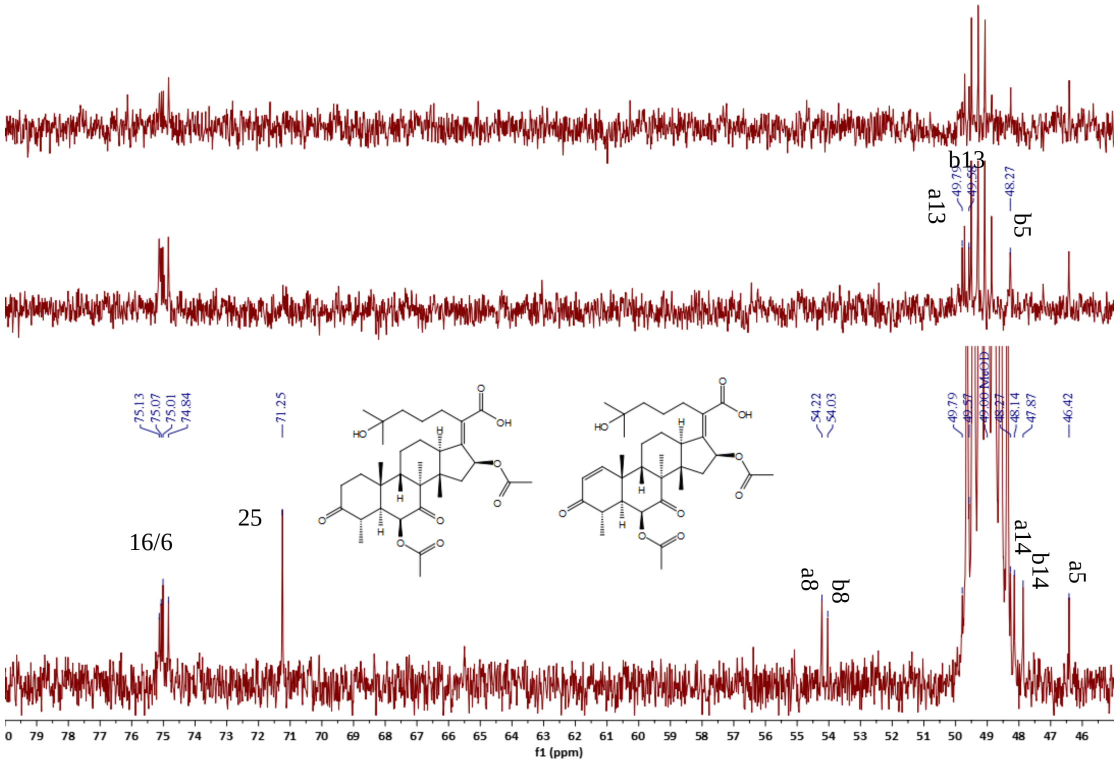

Figure S17. Expansion ^{13}C NMR and DEPT spectrum of compound **3a** and **3b** in CD_3OD

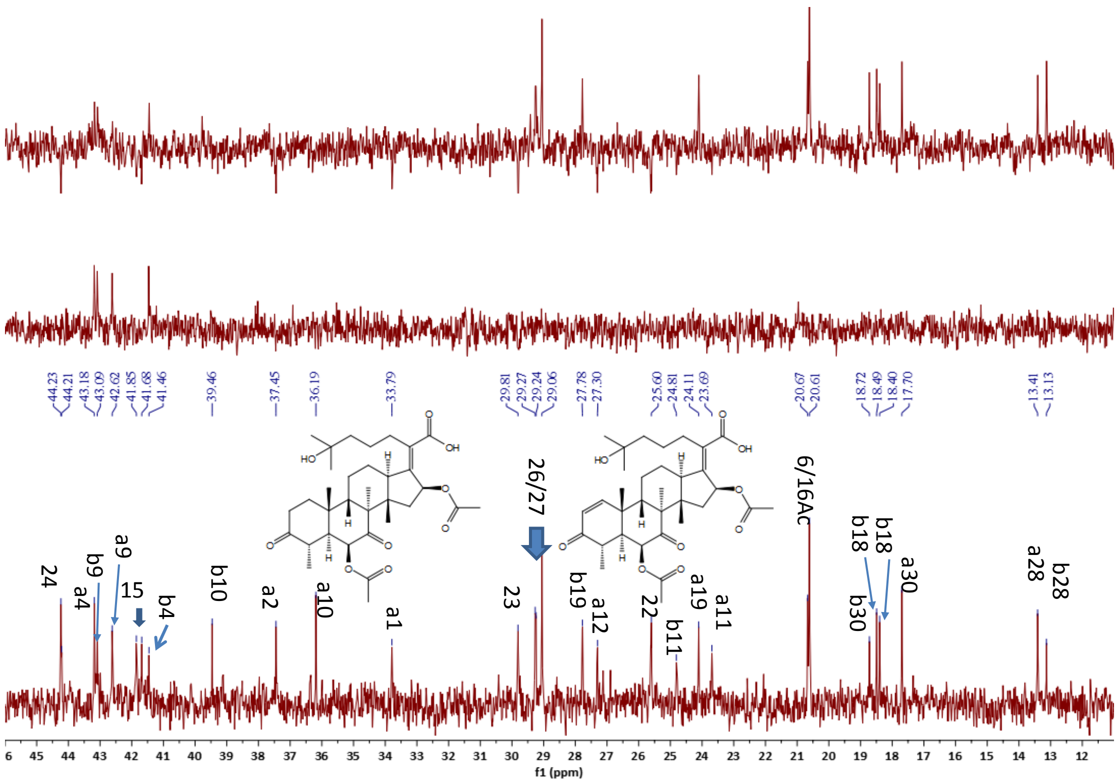

Figure S18. ^1H-^1H COSY spectrum of compound **3a** and **3b** in CD_3OD

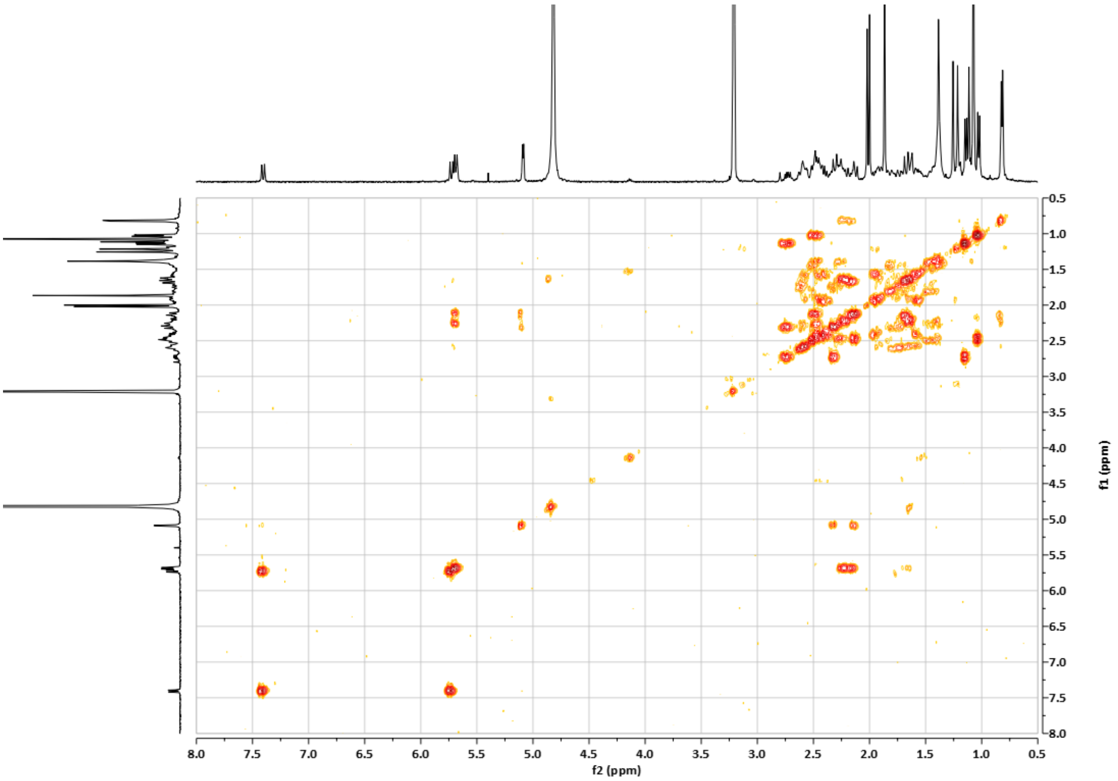

Figure S19. HSQC spectrum of compound **3a** and **3b** in CD_3OD

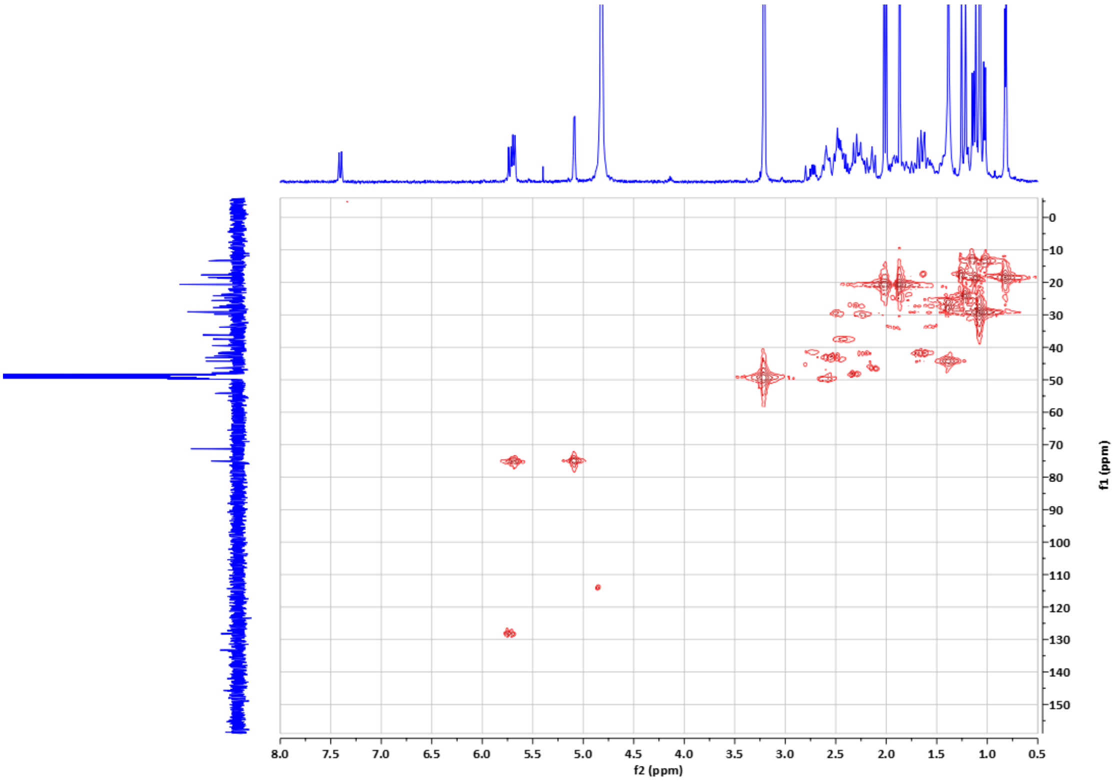

Figure S20. HMBC spectrum of p compound **3a** and **3b** in CD_3OD

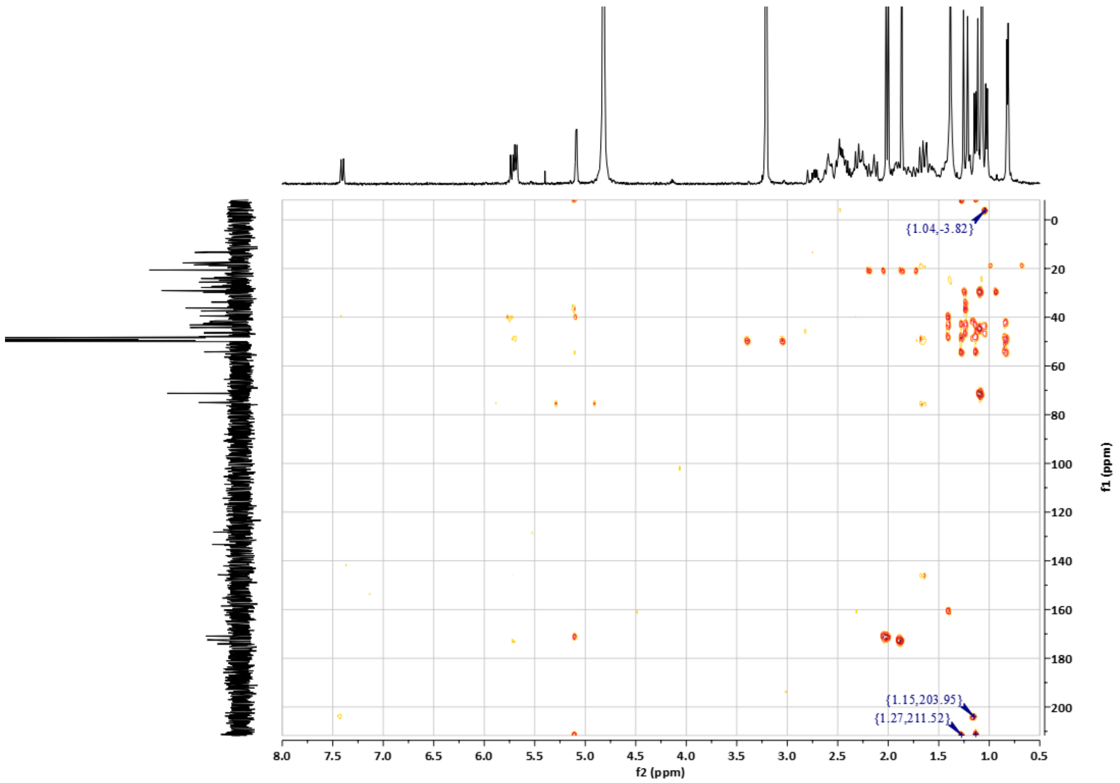

Figure S21. NOESY spectrum of compound **3a** and **3b** in CD_3OD

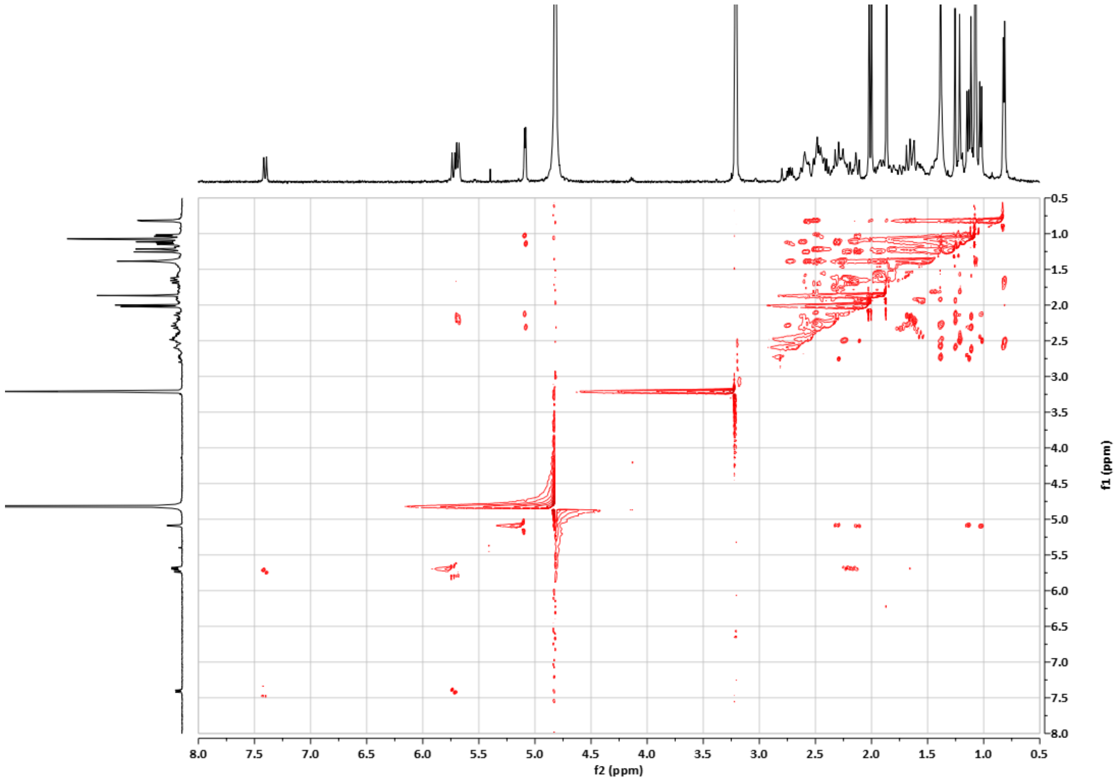

Figure S22. HRESIMS spectrum of compound **3a** and **3b** in CD_3OD

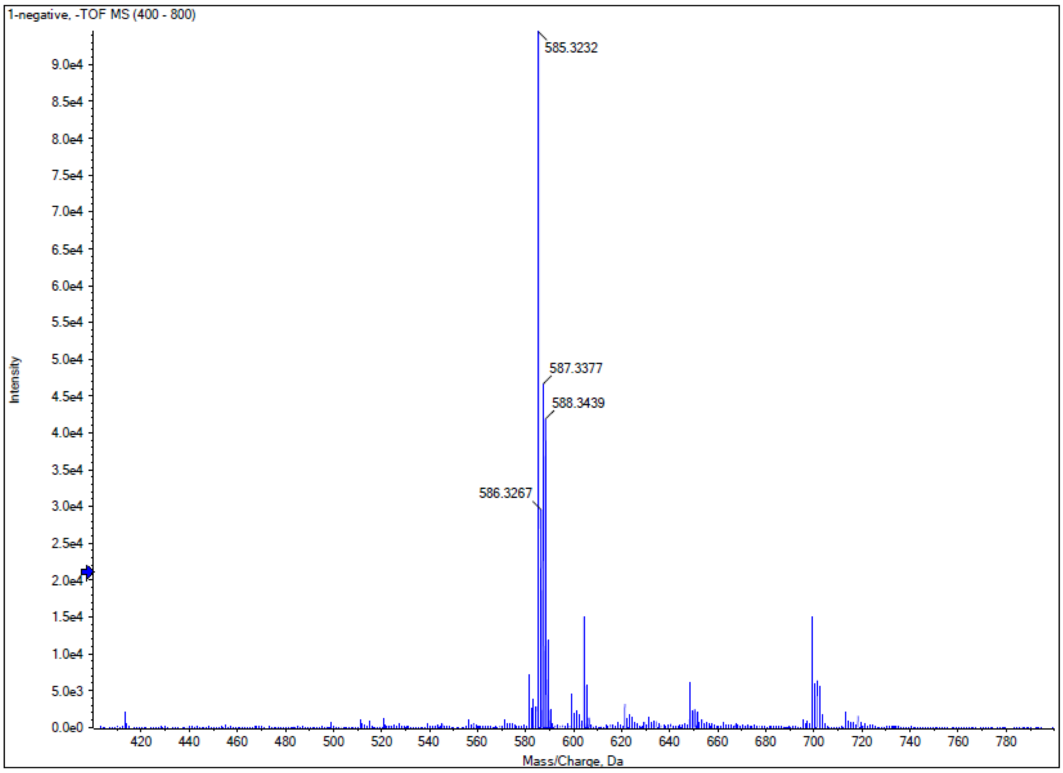

Figure S23. ^{1}H NMR spectrum of compound **5** in $CDCl_3$

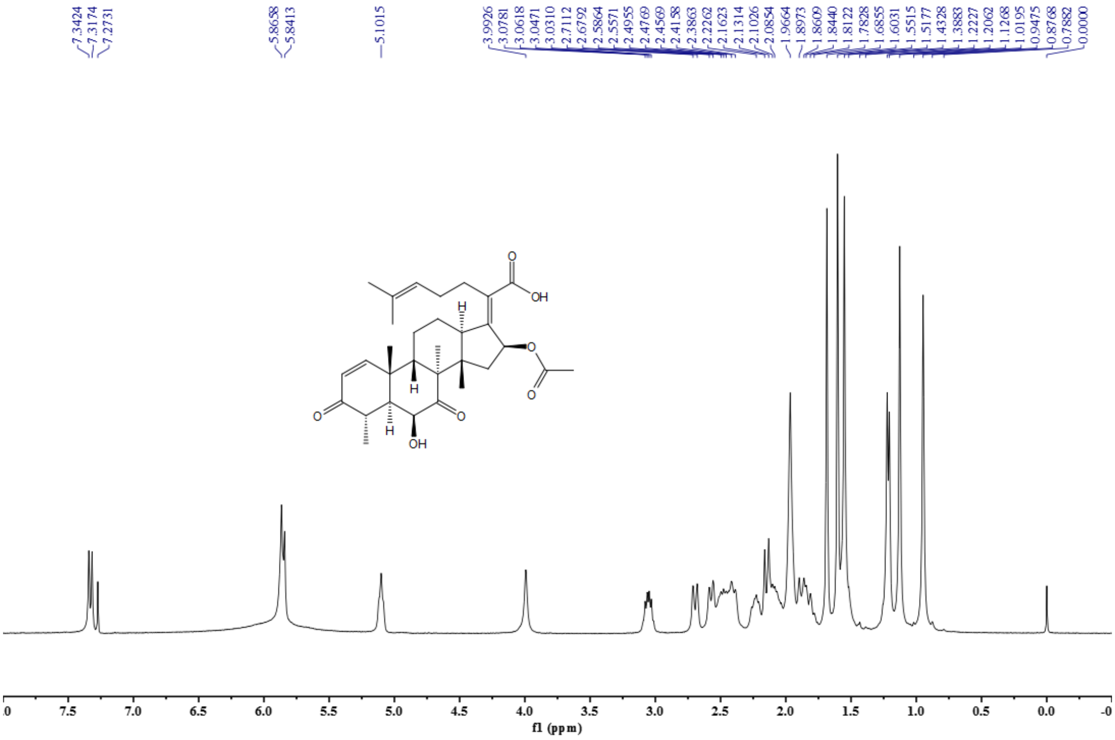

Figure S24. ^{13}C NMR and DEPT spectrum of compound **5** in $CDCl_3$

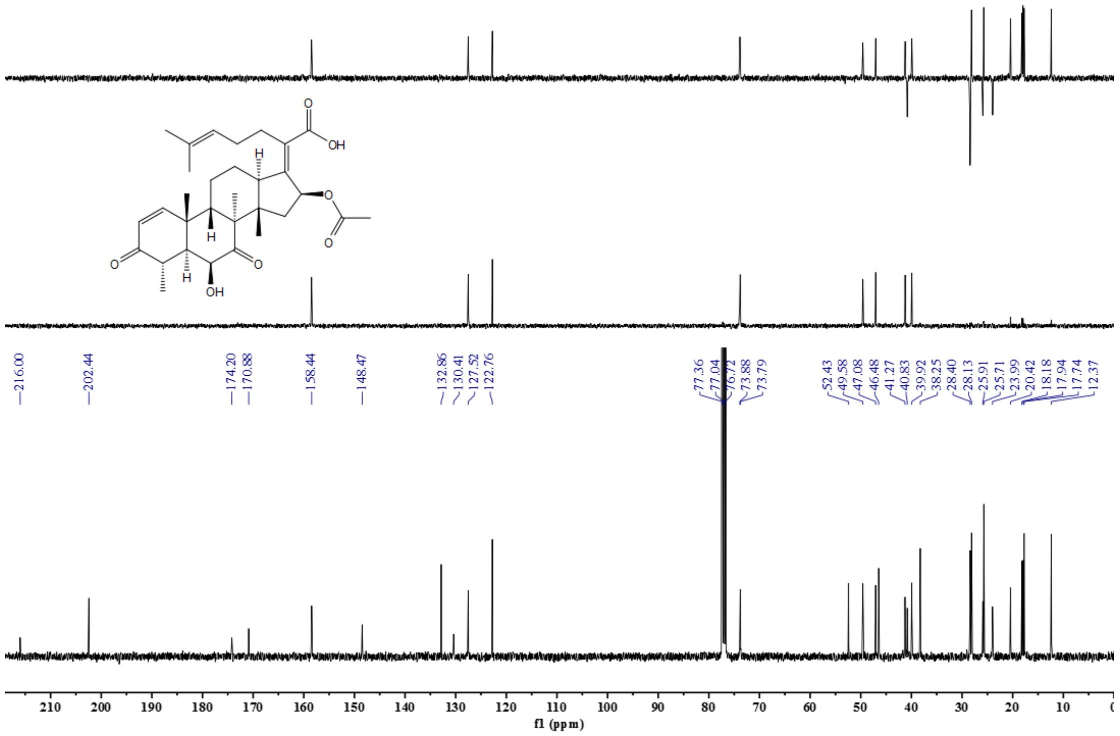

Figure S25. ^{1}H-^{1}H COSY spectrum of compound **5** in $CDCl_3$

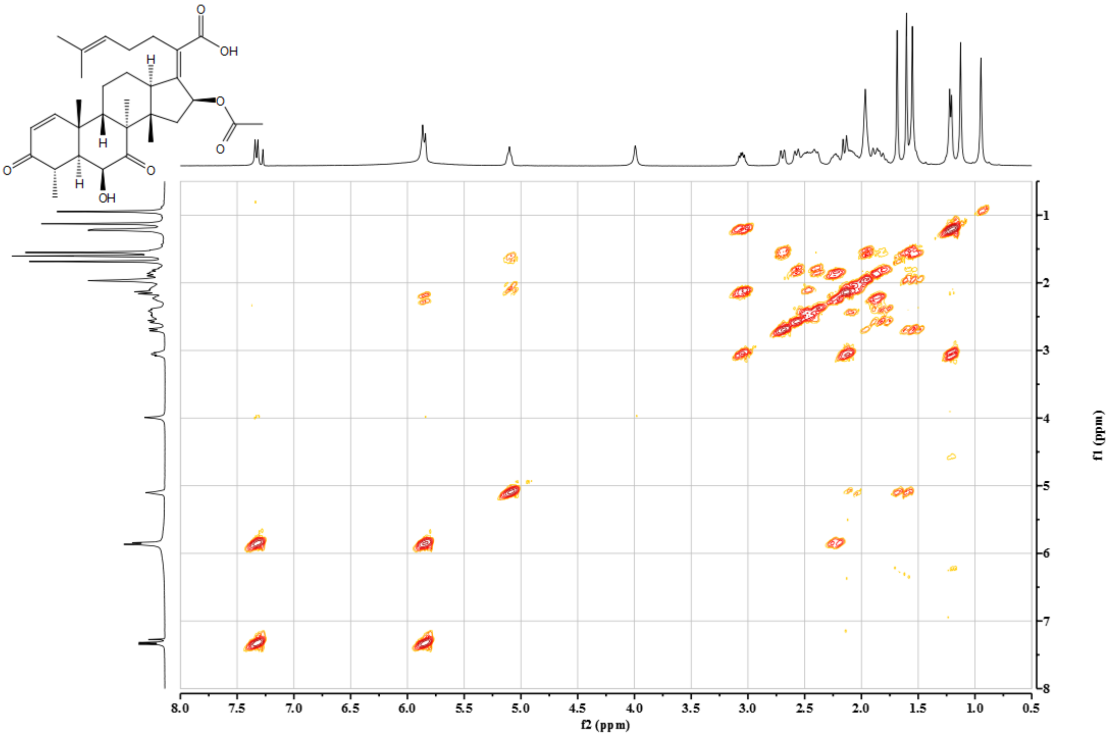

Figure S26. HSQC spectrum of compound **5** in $CDCl_3$

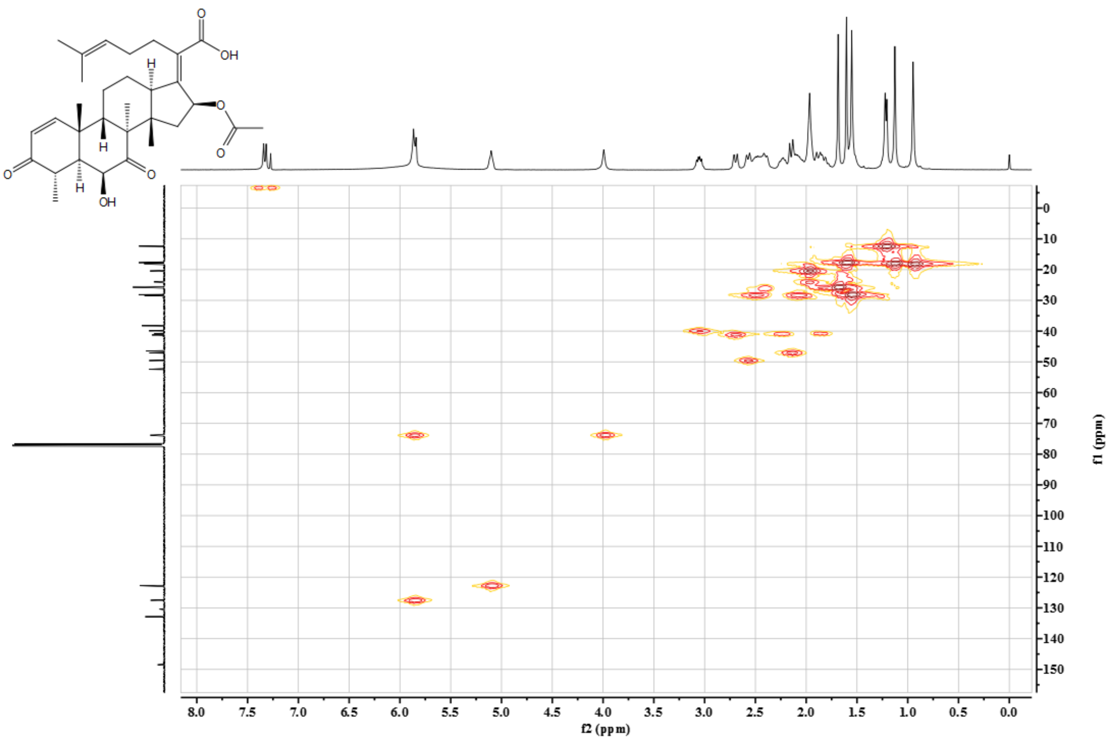

Figure S27. HMBC spectrum of p compound **5** in $CDCl_3$

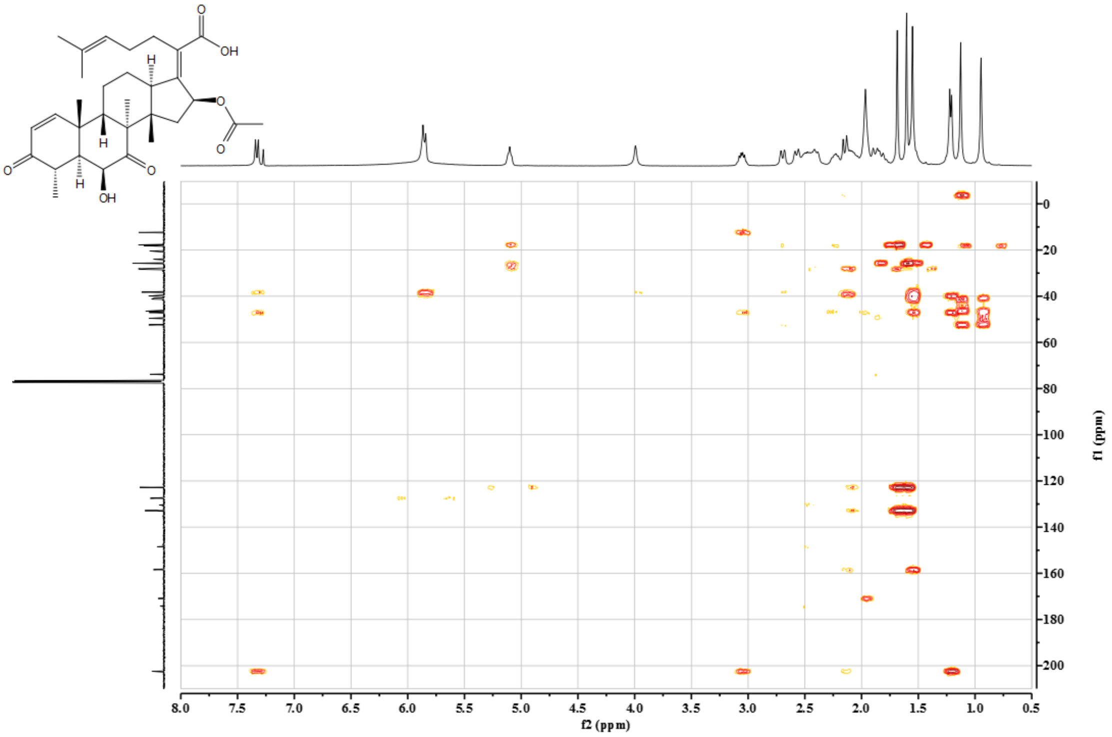

Figure S28. ^{1}H NMR spectrum of compound **6** in $CDCl_3$

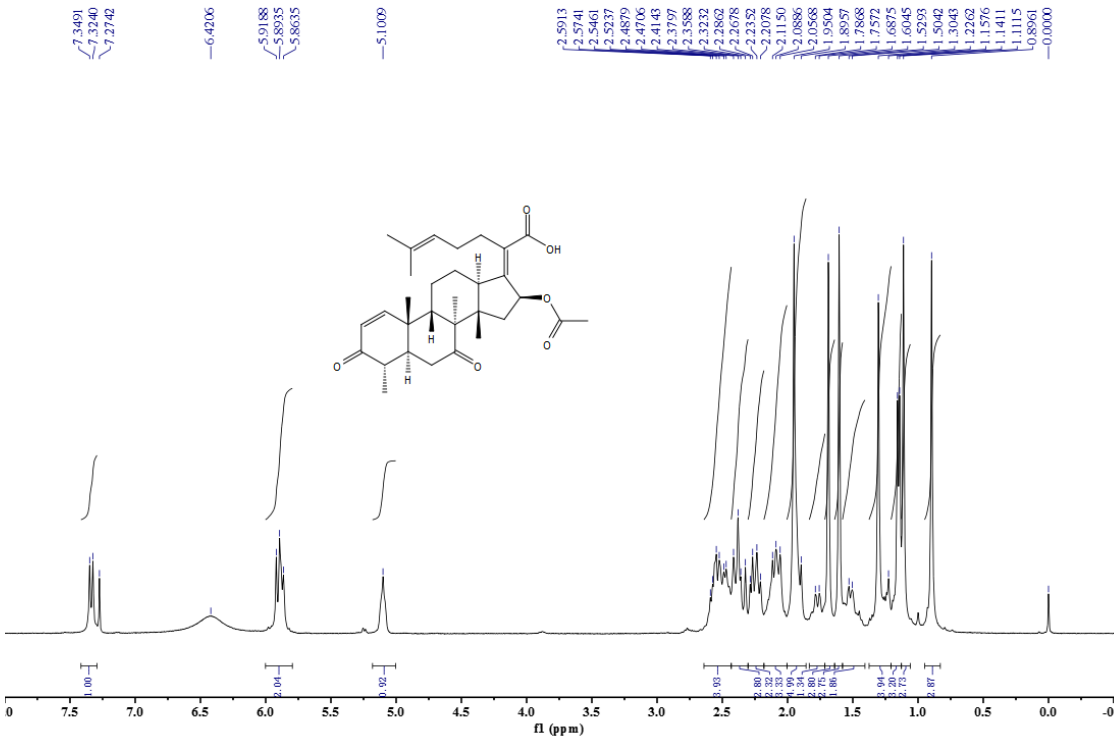

Figure S29. ^{13}C NMR and DEPT spectrum of compound **6** in $CDCl_3$

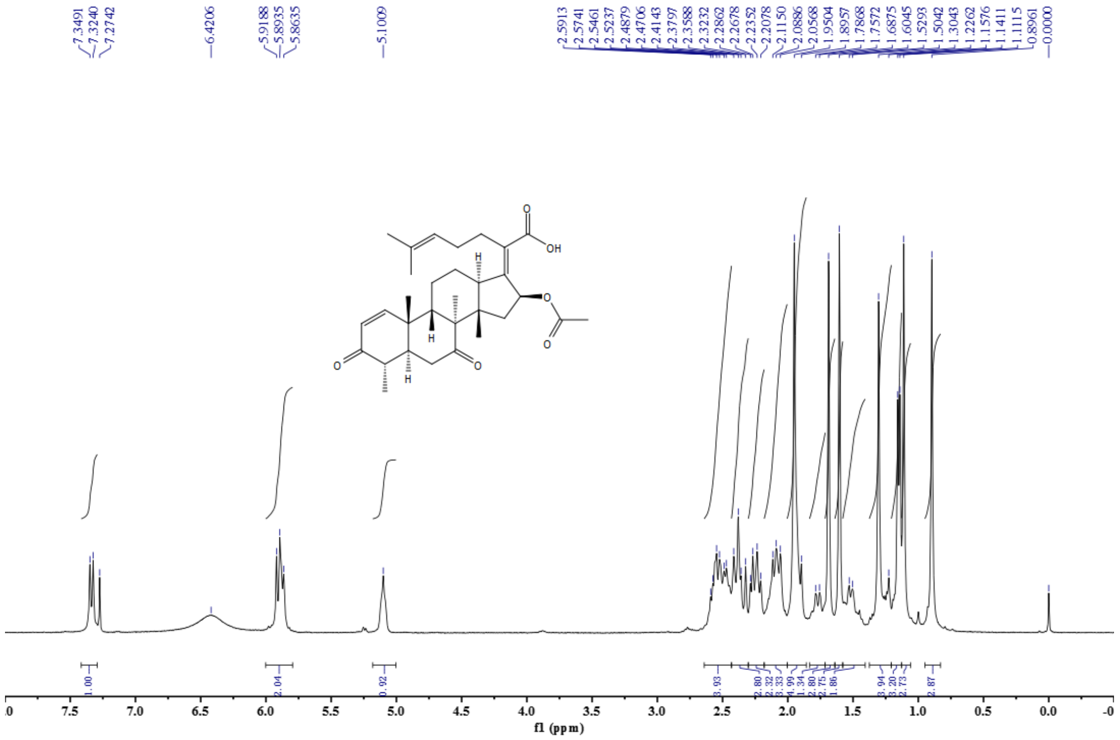

Figure S30. ^{1}H-^{1}H COSY spectrum of compound **6** in $CDCl_3$

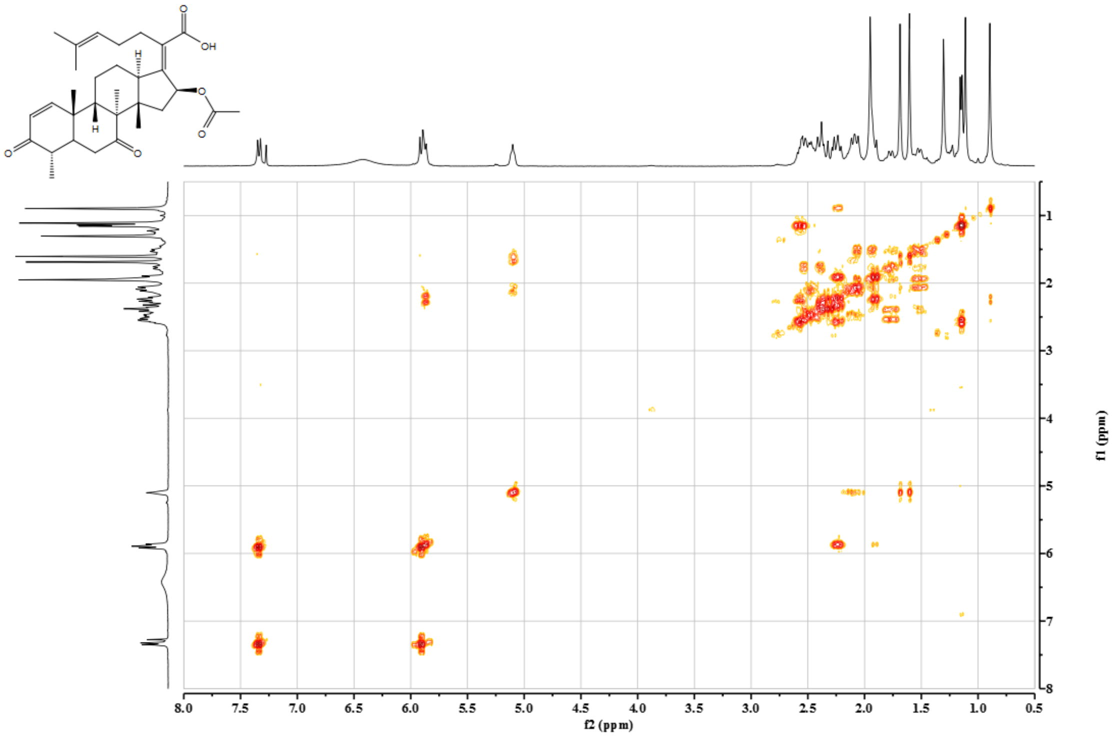

Figure S31. HSQC spectrum of compound **6** in $CDCl_3$

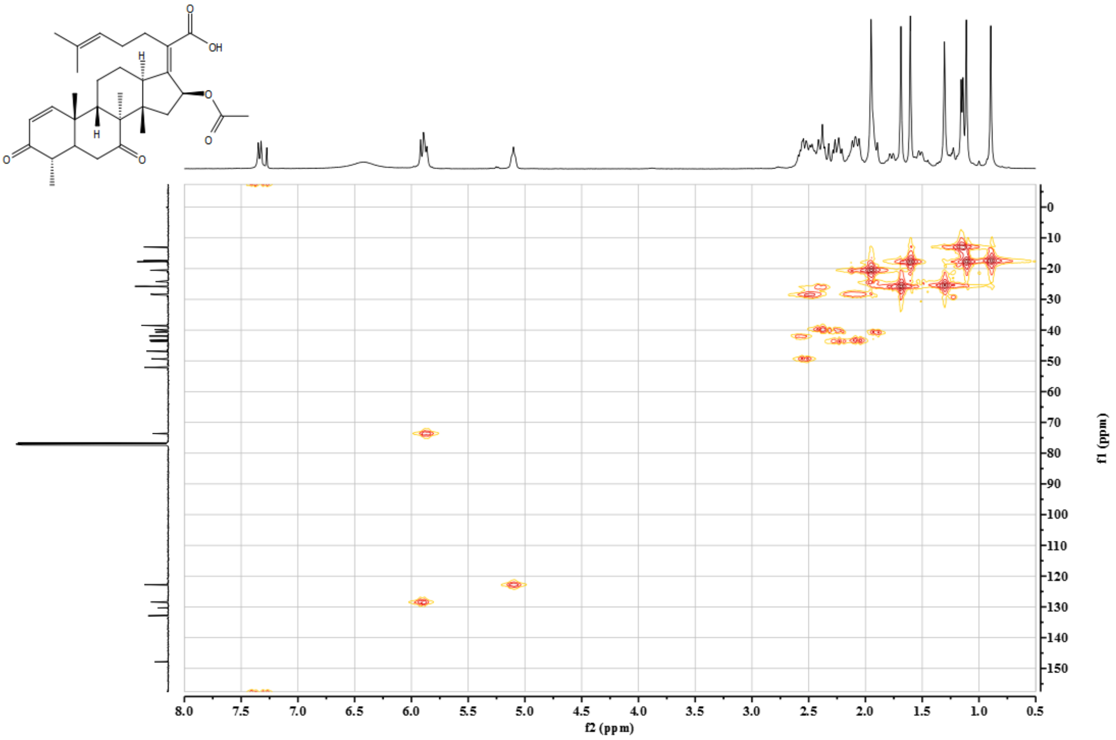

Figure S32. HMBC spectrum of compound **6** in $CDCl_3$

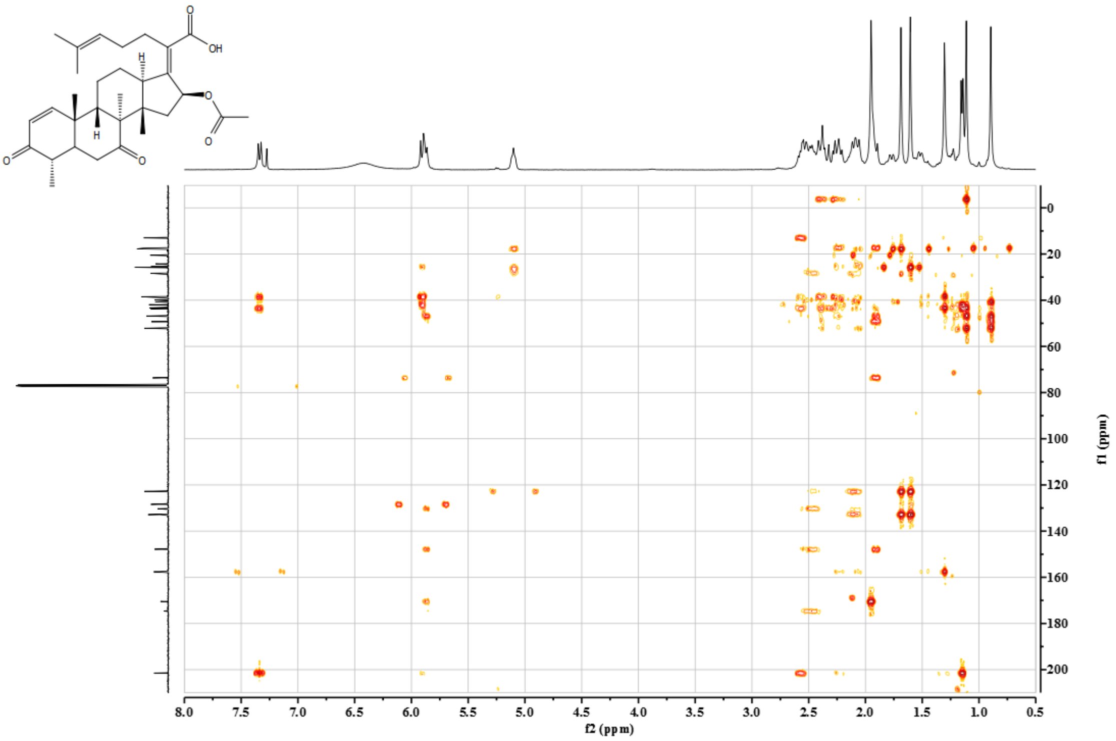

Figure S33. ^{1}H NMR spectrum of compound **7** in CD_3OD

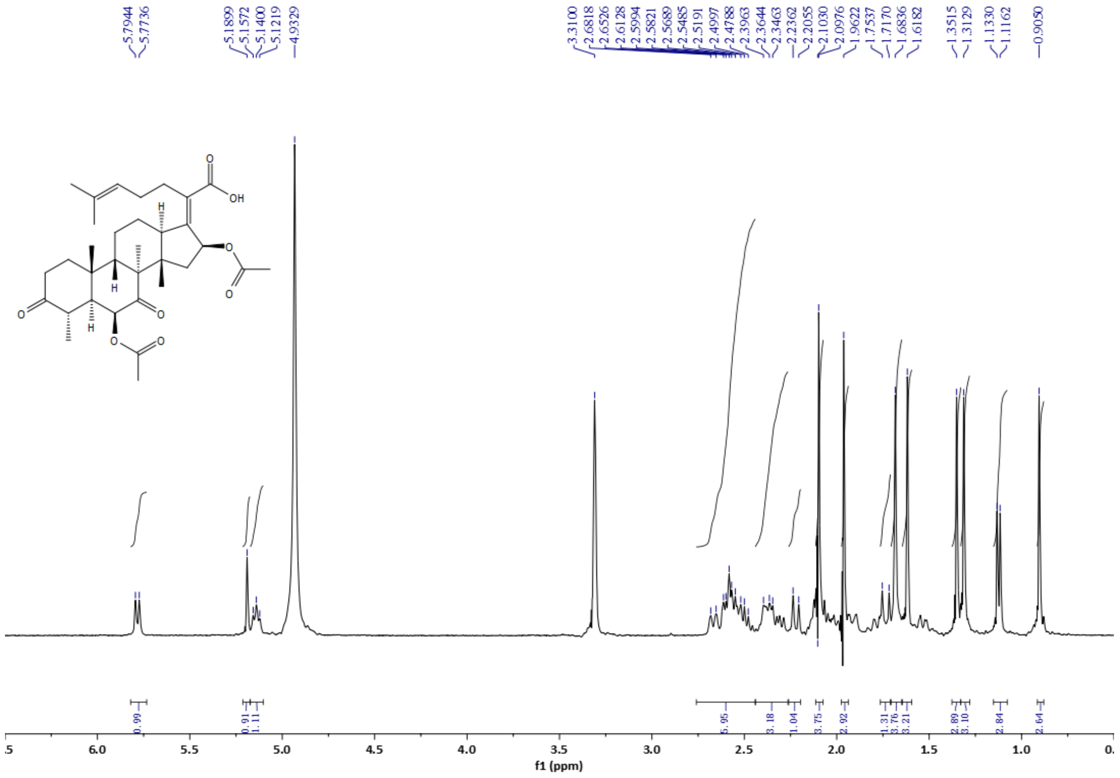

Figure S34. ^{13}C NMR and DEPT spectrum of compound **7** in CD_3OD

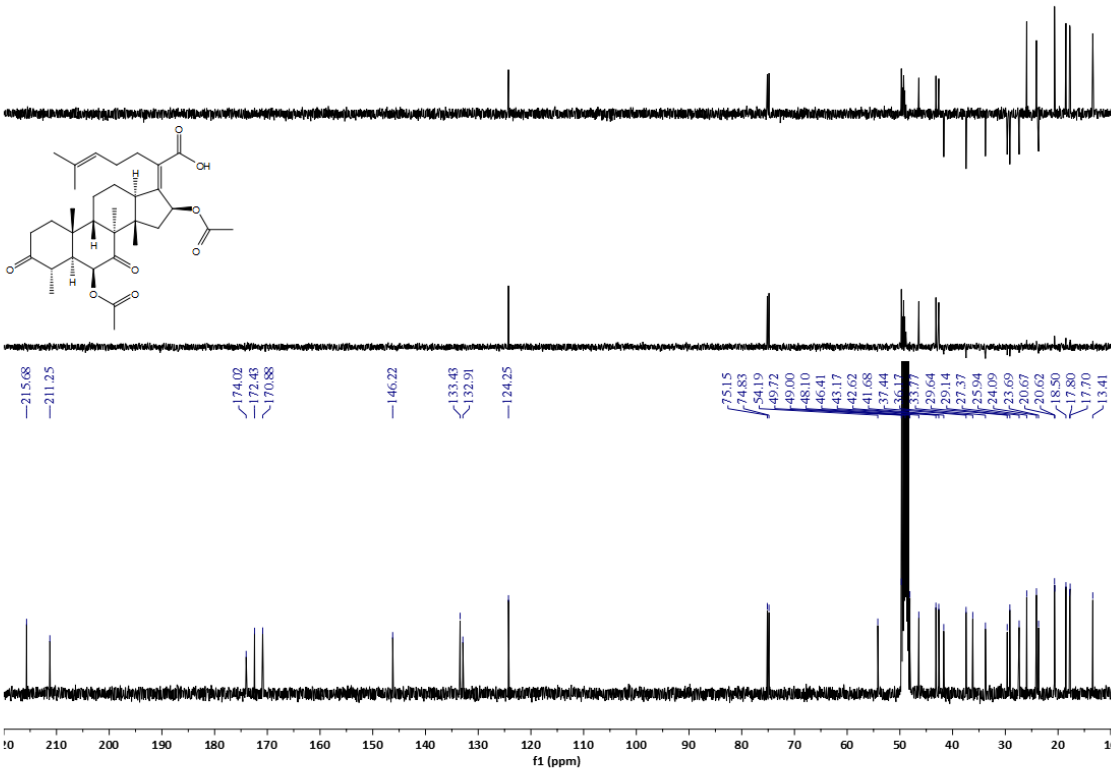

OH
215.68
211.25
174.02
172.43
170.88
146.22
133.43
132.91
124.25
75.15
74.83
54.19
49.72
49.00
48.10
46.41
43.17
42.62
41.68
37.44
36.17
33.77
29.64
29.14
27.37
25.94
24.09
23.69
20.67
20.62
18.50
17.80
17.70
13.41
210 200 190 180 170 160 150 140 130 120 110 100 90 80 70 60 50 40 30 20
f1 (ppm)
